# Electronic Blockade of Shunting Pathways via Dual Insulator Contacts for High-Efficiency Wide-Bandgap Perovskite Indoor Photovoltaics

**DOI:** 10.1007/s40820-026-02225-5

**Published:** 2026-05-19

**Authors:** Quanxi Liu, Yousheng Wang, Qiaoyan Ma, Jianzha Zheng, Yinghui Peng, Liwei Wang, Zeyu Chen, Tianhao Du, Daxin Xiao, Jiandong Fan, Yoon-Bong Hahn, Yaohua Mai

**Affiliations:** 1 Institute of New Energy Technology, College of Physics & Optoelectronic Engineering, Jinan University, Guangzhou, 510632 People’s Republic of China; 2 Department of Electronic Engineering, College of Information Science and Technology, Jinan University, Guangzhou, 510632 People’s Republic of China; 3 Guangdong Mellow Energy Co., Ltd, Zhuhai, 519000 People’s Republic of China; 4 Institute of Applied Physics and Materials Engineering, University of Macau, Macao, 999078 People’s Republic of China; 5 School of Semiconductor and Chemical Engineering, Solar Energy Research Center, Jeonbuk National University, 567 Baekjedaero, Deokjin-Gu, Jeonju-Si, Jeollabuk-Do 54896 Republic of Korea

**Keywords:** Indoor photovoltaics, Wide-bandgap perovskites, Dual insulator contacts, Shunting paths, Defect passivation

## Abstract

**Highlights:**

- Dual insulator contacts integrating poly(methyl methacrylate) (PMMA)-based grain-boundary passivation and a hybrid PMMA/mesoporous alumina ultrathin composite buried-interface layer.
- Dual insulator contacts suppress shunting paths and nonradiative recombination in both the wide-band gap (WBG)-perovskite bulk and heterointerfaces.
- The WBG-Perovskite indoor photovoltaics deliver a power conversion efficiency (i) of 44.36% (power output: 127.94 µW cm^−2^), with a high open-circuit voltage of 1.091 V and fill factor of 83.97% under 1000 lx light-emitting diode (288.4 µW cm^−2^, 2950 K).

**Abstract:**

Perovskite indoor photovoltaics (PIPVs) present great potential for powering Internet-of-Things (IoT)-integrated portable and low-power-consumption wireless electronics, owing to their theoretically high indoor power conversion efficiency (PCE(i)) under natural/artificial lighting conditions. Under low-light irradiance, suppressing shunting paths in PIPVs are of paramount importance to minimize interface defects induced losses in open-circuit voltage (*V*_oc_) and fill factor (FF). In this work, we propose a dual insulator contact (DIC) strategy that synergistically mitigates nonradiative recombination and shunting losses: (i) a grain boundary insulator contact (GIC) using insulating polymer poly(methyl methacrylate) (PMMA) to passivate grain boundary defects and (ii) a buried interface insulator contact (BIC) employing a hybrid PMMA/mesoporous alumina ultrathin composite interlayer to reduce heterointerface defects at the perovskite/hole transport layer junction. Leveraging this DIC approach, wide-band gap (WBG)-PIPVs achieve a PCE(i) of 44.36% (a power output of 127.94 µW cm^−2^) with a high *V*_oc_ of 1.091 V and FF of 83.97% under a light-emitting diode (LED) (1000 lx, 288.4 µW cm^−2^, and 2950 K). Remarkably, the devices under LED illumination remain high indoor performance with small *V*_oc_ and FF deficits even under lower irradiance, delivering PCEs(i) of 43.08% (*V*_oc_ of 1.064 V and FF of 84.52%) at 600 lx, 40.24% (*V*_oc_ of 1.050 V and FF of 82.97%) at 400 lx, and 40.94% (*V*_oc_ of 1.020 V and FF of 82.83%) at 200 lx. The unencapsulated WBG-PIPVs also exhibit robust operational stability under continuous maximum power point tracking in under ambient LED lighting conditions, underscoring their practicality for IoT applications.

**Figure:**
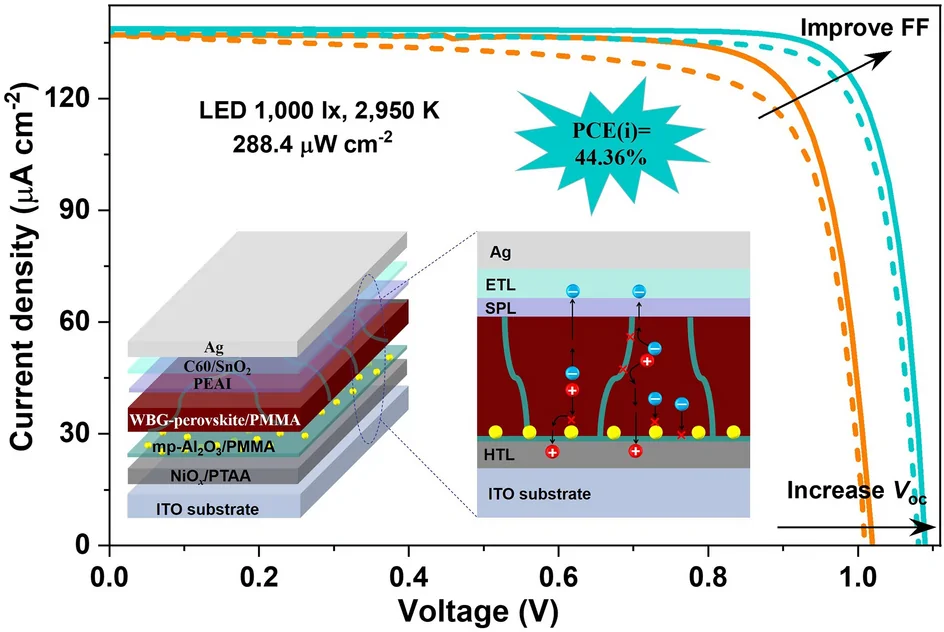

**Supplementary Information:**

The online version contains supplementary material available at 10.1007/s40820-026-02225-5.

## Introduction

Indoor photovoltaics (IPVs), which harness natural/artificial light to generate electricity, offer a sustainable and eco-friendly solution for powering the exponentially increasing number of Internet-of-Things (IoT)-based device electronics [1–3]. Recently, emerging IPV technologies, such as dye-sensitized solar cells (DSSCs), organic photovoltaics (OPVs), and perovskite solar cells (PSCs) have offered significant potential for the development of high-throughput, cost-effective, flexible, and high-performance IPVs [4–7]. Among them, perovskite-based IPVs (PIPVs) have demonstrated particularly remarkable progress through precursor additive engineering [8, 9] and/or device configuration design [7, 10, 11], with most reported indoor power conversion efficiency (PCE(i)) values now exceeding 42% under light-emitting diodes (LEDs) or fluorescent lamps (FLs) illumination. Beyond performance, a life-cycle assessment (LCA) of lead-based PIPVs by Pecunia et al. further underscores their significant potential for environmental sustainability [12]. Notably, within just a decade of development, PIPVs under U30 fluorescent lamp illumination have reached the highest PCE(i) of 44.72% (a record power output of 151.24 µW cm^−2^) with an open-circuit voltage (*V*_oc_) of 1.069 V and fill factor (FF) of 82.3% [13]. Nevertheless, there remains a notable efficiency gap when compared with the maximum theoretical PCEs(i) of PIPVs up to 55.62% and 59.76% under light-emitting diode (LED, 1000 lx, 3000 K, and 301.9 µW cm^−2^) and fluorescent lighting (U30, 3000 K, and 338.2 µW cm^−2^) illumination, respectively [14].

Under low irradiance conditions, the photocurrent is extremely low, resulting in a relatively insignificant impact of series resistance (*R*_s_) due to the limited *V*_oc_ drop across it. Conversely, the influence of shunt resistance (*R*_sh_) becomes increasingly prominent as irradiance decreases, particularly under lower light intensities [3]. Thus, significant reductions in *V*_oc_ and FF are observed under low-light intensities, as excessively low *R*_sh_ leads to substantial leakage currents and increased nonradiative recombination [15, 16]. As is well known, solution-processed polycrystalline wide-band gap (WBG) perovskite films present undesirable interfacial defects during the crystal growth process, including surface and/or buried interface imperfections (such as pin-holes and voids), grain boundaries (GBs) defects, and point defects (particularly undercoordinated Pb and/or halide ions) [17–21]. These film morphological and structural imperfections serve as primary recombination centers, significantly reducing *R*_sh_ and increasing nonradiative recombination in WBG-PIPVs by shunting paths [22–24]. Maintaining high *V*_oc_ and FF values is essential for minimizing the PCE(i) losses in WBG-PIPVs. Thus, the passivation of nonradiative recombination centers at GBs and interfaces of perovskite films is crucial for enhancing the *V*_oc_ and FF of WBG-PIPVs by reducing ohmic leakage currents. Insulator surface and/or interface passivation by using high-resistivity mesoporous oxide nanoparticles [25–27], and a thin dielectric layer or multifunctional polymers/additives [28–32] have been considered as effective methods to heal GBs, improve interface contacts, and suppress nonradiative recombination through the inhibition of shunting pathways. However, the inclusion of a thicker insulator layer at the heterointerface generally results in an increased *R*_s_, leading to a vast FF reduction due to extreme sensitivity of electric tunneling to the dielectric thickness [33, 34].

To address these challenges, we demonstrate a dual insulator contact (DIC) strategy that synergistically combines grain boundary insulator contacts (GIC) and buried interface insulator contacts (BIC), for providing an optimal trade-off between passivation and charge extraction/transport in WBG-perovskite films and at their related interfaces. This DIC strategy significantly reduces nonradiative recombination at both bulk phases (GBs/voids) and the WBG-perovskite/hole transport layer (HTL) heterointerfaces through localized insulator contacts by polymer poly(methyl methacrylate) (PMMA) and mesoporous alumina (mp-Al_2_O_3_), thereby reducing shunting losses in PIPVs. As a result, WBG-PIPVs under LED illumination with a power intensity of 288.4 µW cm^−2^ (1000 lx, 2950 K) show a PCE(i) of 44.36% (a power output of 127.94 µW cm^−2^), accompanied by an impressive *V*_oc_ of 1.091 V and FF of 84.97%. Equally importantly, the devices retain exceptional indoor performance even at lower irradiance conditions under LED illumination, achieving high PCEs(i) of 43.08% (*V*_oc_ of 1.064 V and FF of 84.52%) at 600 lx, 40.24% (*V*_oc_ of 1.050 V and FF of 82.97%) at 400 lx, and 40.94% (*V*_oc_ of 1.020 V and FF of 82.83%) at 200 lx. Our WBG-PIPVs also exhibit excellent operational stability, maintaining over 97% (unencapsulated) of their PCEs(i) during 1440h test of maximum power point tracking under LED light illumination (1000 lx) at ambient conditions (relative humidity of 50%–60% and temperature of 25–30 °C).

## Experimental Section

### Materials

Formamidinium iodide (FAI, ≥ 99.99%) was purchased from Greatcell Solar Materials Pty Ltd. Lead iodide (PbI_2_, ≥ 99.99%), lead bromide (PbBr_2_, ≥ 99.99%), methylammonium bromide (MABr, > 99.995%), and Cesium iodide (CsI, ≥ 99.999%) were purchased from e-Light New Materials Co., Ltd. Phenethylammonium (PEAI, > 99.5%), C60 (> 99.5%), and poly[bis(4-phenyl) (2,4,6-trimethylphenyl) amine (PTAA) were purchased from Xi’an p-OLED Corp. N, N-dimethylformamide (DMF, 99.8%), dimethyl sulfoxide (DMSO, > 99.9%), and Al_2_O_3_ dispersion (30 nm, 20 wt% in isopropanol) were purchased from Sigma-Aldrich. Isopropanol (IPA, ≥ 99.9%), chlorobenzene (CB, 99.8%), ethyl acetate (EA, ≥ 99%), and poly(methyl methacrylate) (PMMA) were purchased from Aladdin. Tetrakis(dimethylamino)tin (TDMASn, ≥ 99%) was purchased from Shanghai Oriphant Chemisty Co., Ltd. ITO substrates were purchased from Yingkou OPV Tech New Energy Co., Ltd. NiO_*x*_ target was purchased from ZhongNuo Advanced Material (Beijing) Technology Co., Ltd. Silver (Ag, ≥ 99.995%) was purchased from JiuYue Advanced Materials Technology Co. Ltd. All the reagents were used as received without further purification.

### Perovskite Solutions and Devices Fabrication

#### Preparation of Perovskite Precursor

The FAPbI_3_ solution was prepared by dissolving 1.38 M FAI and 1.5 M PbI_2_ in a mixed solvent of DMF and DMSO (4:1 v/v). Similarly, the MAPbBr_3_ solution was prepared by dissolving 1.38 M MABr and 1.5 M PbBr_2_ in a mixture of DMF/DMSO (4:1 v/v). Furthermore, a 1.5 M CsI solution was obtained by dissolving CsI in pure DMSO solvent. Finally, the 1.5 M perovskite precursor solution was formulated by combining FAPbI_3_, MAPbBr_3_, CsI, and DMSO solutions in a volume ratio of 44:16:3:3 to form 1.71 eV perovskite (Cs_0.05_FA_0.70_MA_0.25_PbI_2.25_Br_0.75_).

#### Devices Fabrication

ITO substrates underwent that a sequential cleaning process were involving ultrasonication in detergent, deionized water, and IPA for 10 min each. After drying with N_2_ gas flow, the substrates were thermally treated at 200 °C for 5 min to remove residual organic materials, followed by a 10 min oxygen plasma treatment. A 20 nm NiO_*x*_ film was deposited by radio frequency (RF) magnetron sputtering at 65 W power and 2 Pa pressure for 20 min, followed by post-annealing in air at 180 °C for 20 min [35]. After oxygen plasma treatment of ITO/NiO_*x*_ films for 2 min, the PTAA solution (2.5 mg PTAA in 5 mL CB) and Al_2_O_3_ solution (1 mL 20 wt% colloidal Al_2_O_3_ was diluted in 50 mL IPA) were spin-coated successively on the ITO/NiO_*x*_ substrates at 4000 rpm and 3500 rpm for 30 s, respectively; after each step was followed by annealing at 120 °C for 10 min. A composite solution of mp-Al_2_O_3_ and PMMA that was prepared by mixing various volume ratios of mp-Al_2_O_3_ to PMMA (0.2 mg mL^−1^ in EA) was spin-coated on the ITO/NiO_*x*_/PTAA substrates at 3500 rpm for 30 s, followed by annealing at 120 °C for 10 min. The perovskite precursor solution was then spin-coated on the both ITO/NiO_*x*_/PTAA/Al_2_O_3_ and ITO/NiO_*x*_/PTAA/Al_2_O_3_-PMMA substrates for 4000 rpm for 40 s. During the final 10 s of spin coating, 240 μL EA or PMMA-EA solution (0.2 mg mL^−1^ in EA) was drop-casted onto the perovskite films, followed by thermal annealing at 100 °C for 20 min on a hot plate. The PEAI solution (1 mg mL^−1^ in IPA) was coated on the 3D-perovskite surface. Subsequently, a 20 nm C60 layer and 25 nm SnO_2_ layer were deposited by thermal evaporation and atomic layer deposition (ALD), respectively [36]. Finally, 150-nm Ag electrodes were thermally evaporated through a mask to define the active area of 0.09 cm^2^, and an antireflection coating (YK-AR-film-H was purchased from Liaoning Yike Precision) was applied to the rear side of the solar cells.

### Characterization and Measurements

#### Film Characterization

The surface morphology of perovskite films and cross-sectional view of device configuration were examined by field-emission scanning electron microscopy (FE-SEM, FEI Apreo LoVac). The nanoscale surface topography and electrical properties were further measured by tapping-mode conductive atomic force microscopy (C-AFM, Nano Scope NS3A system) and Kelvin Probe Force Microscopy (KPFM). Structural characterization was analyzed using X-ray diffractometer (XRD, Bruker D8 Advance), while the chemical composition and electronic states were analyzed by X-ray photoelectron spectroscopy (XPS, AXIS ULTRA DLD, aluminum Kα X-ray radiation). Upon exciting from the bottom surface near the hole transport layer, the steady-state of perovskite films was analyzed by photoluminescence (PL) and time-resolved photoluminescence (TRPL) spectra using an Edinburgh Instruments FLS980 fluorescence spectrometer with 532 nm excitation source. PL mapping images were obtained by a PL mapping measurement system (Renishaw plc) with a 532 nm excitation source. Photoluminescence quantum yields (PLQYs) of the WBG-perovskite films were measured by using a commercial PLQY measurement system (Edinburgh FLS1000) with a 532 nm light-emitting diode (LED) as the excitation light source. Elemental depth profiling of perovskite films was analyzed by TOF-SIMS (ION-TOF GmbH).

#### Device Measurements

Device performance evaluation was carried out inside a customized black box at ambient air conditions (relative humidity of 50%–60% and temperature of 25–30 °C) without encapsulation [37]. The indoor performance measurements were performed according to previously reported protocols [13, 38–40]. White LED was applied as light sources with a color temperature (T_c_) of 2950 K, and the illumination was calibrated to 200–1000 lx by a spectrometer inside a customized black box. The indoor spectral characteristics and corresponding input power densities of WLED measured using a calibrated GO-2000B spectroradiometer by an independent third-party measurement institute (Sci-go Instrument Testing Platform) of Beijing Keyango Co., Ltd. All the spectral measurements were conducted using a spectroradiometer calibrated within 1 year. The optical resolution of the spectrometer was about 1.1 nm, and the spectral wavelength accuracy was ± 0.6 nm. Before testing *J-V* curves, we first turn on the simulated LED light source until the stable condition of the output power. This preconditioning was essential for spatial uniformity (across multiple areas, light source inhomogeneity (*U*) ≤ 1%) and temporal stability (over 20 min, instability of light source (*S*) ≤ 2%) under 1000 lx illumination. The angular effects were assessed by comparing indoor performance at two different working distances (WDs) under the same illuminance (1000 lx) [41], which confirmed a deviation (*ε*) of less than 5% (see Fig. S1 and Table S1). A baffle plate was further used to achieve homogeneous light penetrability and eliminate any stray lights for ensuring the temporal stability and spatial uniformity of WLED illumination across various light intensities [37–39]. The current density–voltage (*J-V*) curves and performance parameters of PSCs were carefully measured by a Newport solar simulator (Keithley series 2400, ORIEL-SOI3A) under WLED illumination inside a customized black box. The illuminance level was adjusted by varying the distance between the light source and the device. External quantum efficiency (EQE) spectra were measured by using a QE-R instrument from Enlitech, calibrated with a silicon reference cell. All devices with dimensions of 2.5 cm × 2.5 cm were measured through a 0.09 cm^2^ mask/aperture. To understand the electrical and charge transport characteristics of the devices, electronic impedance spectroscopy (EIS) and dynamic *I-V* curves were performed by using a semiconductor analyzer (ZAHNR CIMPS-2).

#### Calculation of Trap Density

The trap density (*N*_t_) can be calculated by following equation:

1 $$N_{{\mathrm{t}}} = \frac{{2V_{{{\mathrm{TFL}}}} \varepsilon_{{\mathrm{r}}} \varepsilon_{0} }}{{{\mathrm{eL}}^{2} }}$$

where ${V}_{TFL}$, ${\varepsilon}_{r}$, ${\varepsilon}_{0}$, e, and L are the trap-filled limit voltage, relative dielectric constant of the perovskite (35), vacuum permittivity, elementary charge, and thickness of the perovskite layer, respectively.

#### Calculation of Ideal Factor

The ideal factor (*n*) can be calculated by following equation:

2 $$n = \frac{q}{{k_{{\mathrm{B}}} T}} \times \frac{{dV_{{{\mathrm{OC}}}} }}{d(\ln (L))}$$

where the *k*_B_ is the Boltzmann constant, *T* is the temperature, *q* is the electric charge, *L* and *V*_oc_ are light intensity and voltage, respectively.

### Theoretical Simulation

All calculations were performed using the CP2K simulation package (version 7.1) within the framework of the density functional theory [42, 43]. The calculations employed a hybrid Gaussian and plan-wave scheme, with molecular orbitals of the valence electrons using DZVP-MOLOPT-SR-GTH basis sets. Electron exchange–correlation interaction was treated using the Predew–Burke–Ernzerhof (PBE) functional, supplemented with the Grimmes D3 dispersion correction. The interaction between the valence electrons and atomic cores was described by the norm-conserving Goedecker–Teter–Hutter (GTH) pseudopotentials. A plane-wave density cutoff of 500 Ry was adopted. All the structures fully relaxed by CP2K with BFGS scheme, and the force convergence criterion was set to 4.5 × 10^−4^ hartree/bhor.

## Results and Discussion

### Device Configuration Design with Dual Insulator Contacts

**Fig. 1:**
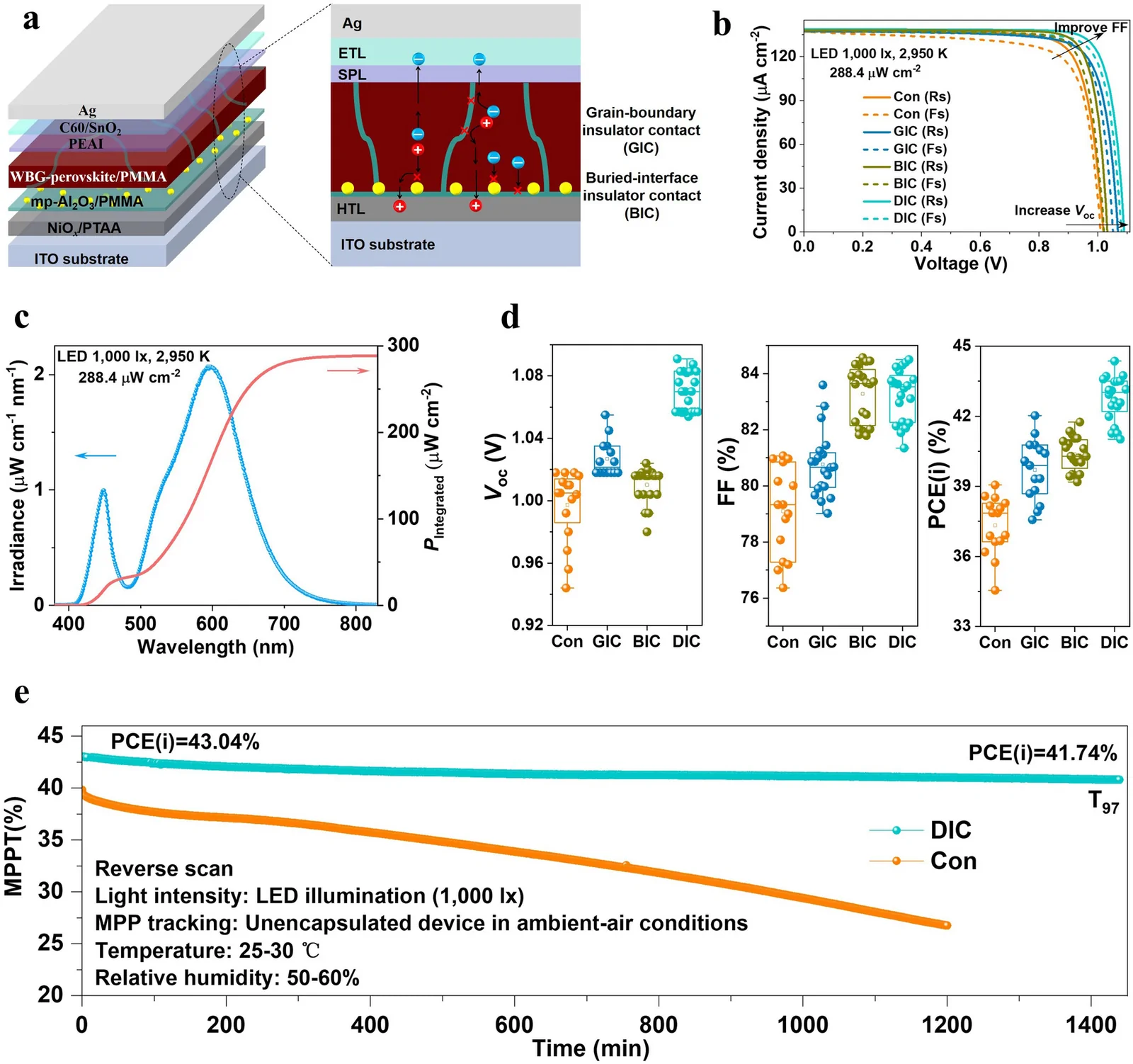
Device configuration and role of dual insulator contacts (DIC) and PIPV characteristics. **a** Wide-bandgap PSC device structure and concept illustration of dual insulator contacts (DIC) for grain boundary insulator contact (GIC) and buried interface insulator contact (BIC). **b** *J-V* curves of optimal performance incorporated reverse and forward scan, and **c** Spectrum of light source under 1000 lx illuminance and the corresponding integrated light source power. **d** performance statistics for four types of WBG-PIPVs measured under LED illumination (1000 lx, 288.4 µW cm^−2^, 2950 K). **e** Operational stability of unencapsulated control and DIC-devices at MPP tracking under LED illumination in ambient air conditions

Figure 1a shows the device structure of ITO/HTL (NiO_*x*_/PTAA)/mp-Al_2_O_3_-PMMA/WBG-perovskite-PMMA/SPL (surface-passivation layer, PEAI)/ETL (C60/SnO_2_)/Ag (left) and related roles of dual insulator contacts (DIC) (right). The DIC is composed of grain boundary insulator contact (GIC) and buried interface insulator contact (BIC) through localized insulator contacts. The GIC architecture is engineered through the strategic incorporation of PMMA polymer insulator at GBs within WBG-perovskite films via an antisolvent-processed approach (for detail, see Experimental Section), which simultaneously promotes the formation of bulk heterojunction structures. This method can facilitate the formation of a perovskite DMSO PMMA (polymeric Lewis base) adduct and heterogeneous nucleation, leading to homogeneous growth of perovskite crystal due to the lower total Gibbs free energy [44], and the PMMA can effectively occupy GBs and/or voids within WBG-perovskite films, ultimately forming WBG-perovskite/PMMA bulk heterojunction structures. Unlike DMSO, which escapes during annealing due to its relatively high vapor pressure, leaving behind voids in perovskite films, the long-chain polymer remains thermally stable throughout the annealing process [45]. Thus, the PMMA insulator can effectively inhibit charge recombination at GBs and/or voids, and thus facilitating charge separation/transport. Furthermore, we systematically uncover significant, previously unreported roles of PMMA in modulating the crystallization dynamics of bromine-rich WBG-perovskites, suppressing of shunting pathways, homogenizing charge distribution and contributing to passivation mechanisms in PIPVs under indoor lighting conditions (for detail, see Sect.  3.3 and 3.4). Due to the similar insulator contact properties of both mesoporous alumina (mp-Al_2_O_3_) and PMMA, the combination of these two materials as a hybrid ultrathin composite interlayer, serving as an integrated functional unit for co-buried interface engineering, is conceptually defined as the BIC. This BIC structure is formed through the integration of a hybrid ultrathin composite interlayer incorporated with mp-Al_2_O_3_ and PMMA polymer at the WBG-perovskite buried interface. The configuration results in a continuous ultrathin PMMA insulator layer on the HTL, which, together with the homogeneous distribution of mp-Al_2_O_3_ (Fig. S3), further improves buried interface contacts and thereby effectively suppresses nonradiative recombination. Furthermore, the presence of homogeneous distribution of incontinuous mp-Al_2_O_3_ in ultrathin hybrid composite layer not only improves the surface wetting quality of hydrophobic PMMA for high-quality deposition of WBG-perovskite precursor (Fig. S2), but also serves as an effective buried interface insulating contact (Fig. S3) facilitating efficient electron blocking and hole transport [46–48]. Thus, by optimizing the mp-Al_2_O_3_-to-PMMA ratio, this hybrid ultrathin composite interlayer serves as the BIC for the WBG-perovskite layer, which not only promotes high-quality perovskite deposition but also reduces recombination rate at the perovskite/HTL heterointerface by effectively inhibiting shunt leakage paths (for detail, see Sect. 3.3).

### Indoor Photovoltaic Performance of WBG-PSCs based on Dual Insulator Contacts

To value indoor performance, we fabricated four types of WBG-PSCs: (i) ITO/HTL/mp-Al_2_O_3_/WBG-perovskite/SPL/ETL/Ag (Control, Con), (ii) ITO/HTL/mp-Al_2_O_3_/WBG-perovskite-PMMA/SPL/ETL/Ag (GIC-device), (iii) ITO/HTL/mp-Al_2_O_3_-PMMA/WBG-perovskite/SPL/ETL/Ag (BIC-device), and (iv) ITO/HTL/mp-Al_2_O_3_-PMMA/WBG-perovskite-PMMA/SPL/ETL/Ag (DIC-device). All devices without encapsulations were measured under LED illumination (1000 lx, 288.4 µW cm^−2^, 2950 K) inside a customized black box at ambient conditions (relative humidity of 50%–60% and temperature of 25–30 °C), with their *J-V* characteristics incorporated reverse and forward scan, and corresponding photovoltaic parameters presented in Fig. 1b and Table S2, respectively. Indoor illumination was provided by commercial WLED lighting with a color temperature (*T*_c_) of 2950 K, and the spectral properties and corresponding integrated power density are presented in Fig. 1c. The influence of PMMA content in the antisolvent on the indoor photovoltaic performance of the GIC-device was systematically optimized, as illustrated in Figs. S4 and S5, Table S3. Note that a high concentration of PMMA may introduce significant electrical resistance, impairing charge transport and consequently reducing both the FF (from 83.93% to 76.22%) and *V*_oc_ (from 1.064 V to 0.944 V), as observed in Table S3 and Fig. S5. Moreover, the influence of the PMMA ratio in mp-Al_2_O_3_ on the indoor photovoltaic performance of the BIC-device was also optimized, as shown in Figs.  S6 and S7, Table S4. Thus, under optimal conditions (PMMA concentration of 0.2 mg mL^−1^ and an Al_2_O_3_:PMMA ratio of 5:1), the GIC-, BIC-, and DIC-devices demonstrate high reproducibility and significant enhancement in *V*_oc_ and FF, thereby improving PCEs(i), compared to the control device. The performance statistics of ~ 20 devices as shown in Fig. 1d and Tables S5–S8 further confirms this conclusion. The improved *V*_oc_ and FF values are mainly attributed to the formation of bulk heterojunction WBG-perovskite films and improved interface contacts, achieved via defects passivation with PMMA and mp-Al_2_O_3_ based localized insulator contacts, thereby suppressing nonradiative recombination (Figs. 3 and S16–S19). In comparison, PIPVs passivated with a single component, i.e., mp-Al_2_O_3_ only (control), GIC or BIC, still exhibited relatively lower indoor performance, particularly in terms of *V*_oc_ or FF, further confirming the critical role of suppressing shunt leakage paths across both WBG-perovskite bulk phases and their interfaces under indoor lighting conditions.

As a result, the champion DIC-device combined with GIC and BIC strategies shows reduced *J-V* hysteresis behaviors and an improved PCE(i) of 44.36% (reverse scan, and a power output of 127.94 µW cm^−2^) with a high *V*_oc_ of 1.091 V, *J*_sc_ of 138.72 μA cm^−2^ and FF of 83.97%. The DIC-device exhibits markedly reduced *J-V* hysteresis, with a hysteresis index (HI) value of 0.019 compared to 0.070 for the control device, indicating that the insulating contacts effectively inhibit ion migration, nonradiative recombination and current shunting [45]. The DIC-devices also show a steady-state power output (SPO) of 42.14% and a *J*_sc_ of 128.74 μA cm^−2^, measured at the maximum power point (MPP) voltage of 0.944 V for 1200 s (Fig. S8). Further integrating the external quantum efficiency (EQE) spectrum (Fig. S9) with the 1000 lx LED spectrum (Fig. 1c) yields a calculated *J*_sc_ of 131.41 μA cm^−2^, which is in close agreement with the measured *J*_sc_ of 138.72 μA cm^−2^ under 1000 lx illumination, with only a 5% discrepancy. Furthermore, compared to control devices, our unencapsulated DIC-PIPVs also showed excellent operational stability, maintaining over 97% of their PCEs(i) during 1440 min test of MPP tracking under LED light illumination (1000 lx, 288.4 µW cm^−2^, 2950 K) at ambient conditions (relative humidity of 45%–50% and temperature of 25–30 °C) (Fig. 1e).

**Fig. 2:**
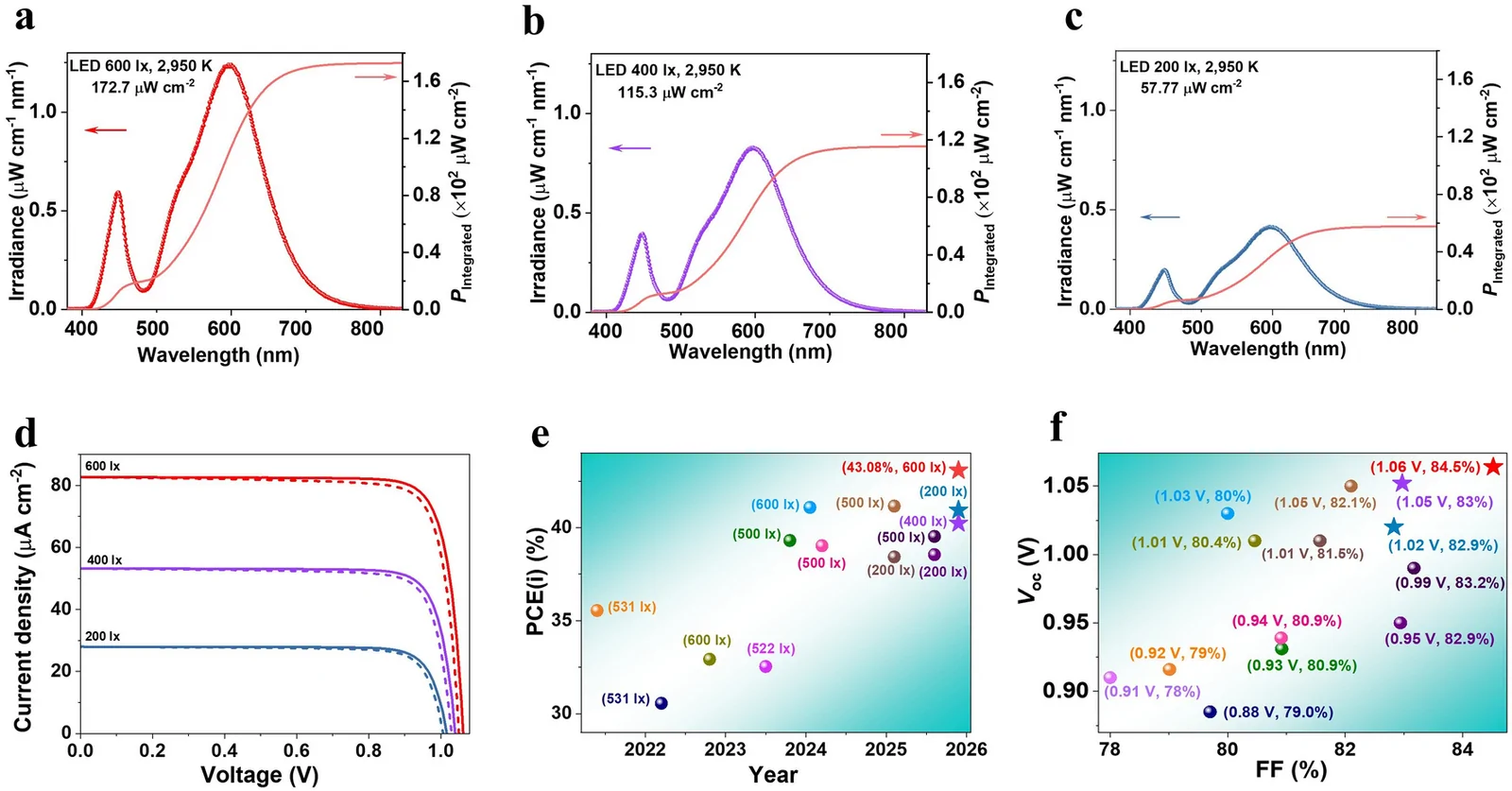
Indoor characteristics for dual insulator contacts (DIC)-based WBG-PIPVs under lower irradiances. **a**–**c** Spectrum of light source under LED illuminance intensities (600 lx, 400 lx and 200 lx) and the corresponding values of integrated light source power. **d** *J-V* curves incorporated reverse (solid lines) and forward (dash lines) scans of the champion DIC-device under low illumination with different light irradiances. **e** Summarized PCEs(i) of representative studies from 2021 to 2025, and **f** corresponding *V*_oc_ versus FF of the PIPVs under low illumination (~ 600 lx, LED)

For typical indoor lighting conditions, illuminance levels generally range from 200 lx in residential living spaces to 300–500 lx in office environments [49]. Thus, we further evaluated the advantages of the DIC-based strategy in suppressing leakage currents and nonradiative recombination, enabling high-performance WBG-PIPVs even under lower irradiance conditions (~ 600 lx). The LED spectrum was also characterized under dark chamber at varying illumination intensities of 600, 400, and 200 lx, corresponding to input power densities of 172.7, 115.3, and 57.77 µW cm^−2^, respectively, as illustrated in Fig. 2a–c. Remarkably, despite the significantly reduced indoor light intensities, the DIC-PIPVs exhibit excellent retention of both *V*_oc_ and FF values over 1.01 V and 81%, respectively, yielding to high PCEs(i) exceeding 40% in both reverse and forward scan directions, as shown in Fig. 2d and Table 1. It should be noted that the integrated *J*_sc_ values derived from the EQE spectra (Fig. S9) with the related LED spectra (Fig. 2a–c) show close agreement with the actual *J*_sc_ values measured under various illuminance intensities of 600, 400, and 200 lx (Fig. S10). To further underscore the capability of the DIC strategy in significantly suppressing shunting pathways in PIPVs, the device was also evaluated under 50 lx illumination, achieving a high PCE(i) exceeding 37% while maintaining a *V*_oc_ of 0.94 V and an FF of 81.08% (Fig. S11). These results further indicate the great potential of DIC-PIPVs for actual applications in low-power-consumption IoT-based wireless protocols such as RFID, LoRa, BLE, ANT, and Zigbee, operating under ambient natural/artificial light conditions (200–600 lx) [50, 51]. To provide a comprehensive performance comparison across different types of LED lighting conditions, we further summarized the reported PCE(i) values along with their corresponding *V*_oc_ and FF values under weak irradiance conditions (200–600 lx, LED illumination), as presented in Fig. 2e, f and Table S9, clearly showing the superior indoor photovoltaic performance in our DIC-PIPVs. Note that indoor performance is also significantly influenced by the input power density, color temperature, and color rendering index, highlighting the importance of lighting choices for PIPV deployment in real-world environments [52, 53].

**Table 1:** Photovoltaic parameters of DIC-PIPVs under light-emitting diode illumination with low irradiances for both reverse and forward scan (*R*_s_ and *F*_s_)

Scan direction	*P*_in_ (μW cm^−2^)	PCE(i) (%)	*V*_oc_ (V)	*J*_sc_ (μA cm^−2^)	FF (%)	*P*_out_ (μW cm^−2^)
600 lx-Rs	172.7	43.08	1.064	82.74	84.52	74.41
600 lx-Fs	172.7	42.15	1.052	82.70	83.67	72.80
400 lx-Rs	115.3	40.24	1.050	53.25	82.97	46.40
400 lx-Fs	115.3	39.42	1.040	53.22	82.13	45.45
200 lx-Rs	57.77	40.94	1.020	27.99	82.83	23.65
200 lx-Fs	57.77	39.77	1.010	27.99	81.28	22.98

### Film Homogeneity and Recombination Behavior by Dual Insulator Contacts

**Fig. 3:**
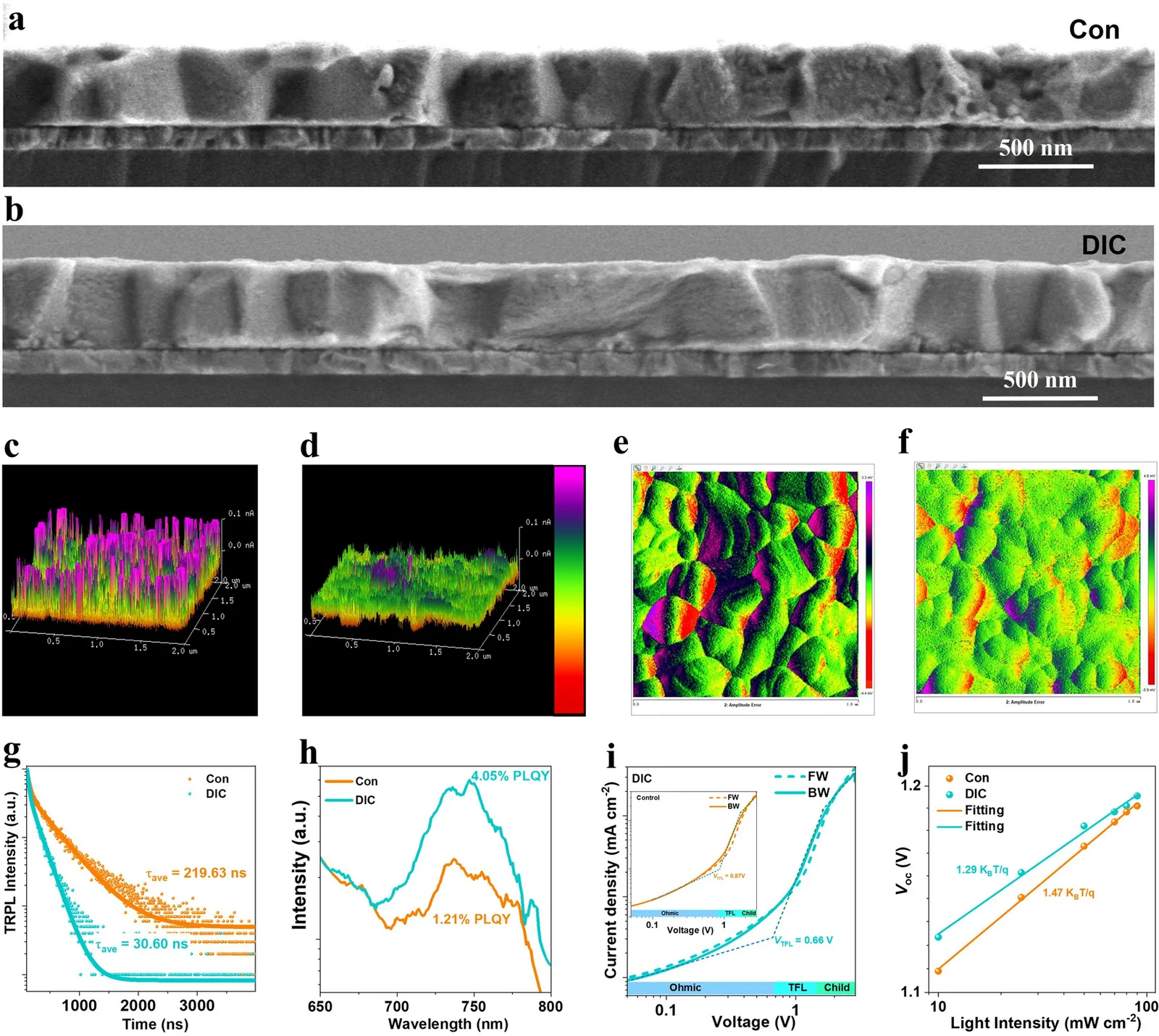
Characteristics of WBG-perovskite films treated with dual insulator contacts (DIC). **a, b** Cross-sectional SEM images, **c, d** C-AFM images, **e, f** KPFM images, **g, h** TRPL spectra and PLQY for control (ITO/NiO_*x*_/PTAA/Al_2_O_3_/WBG-perovskite) and DIC-based WBG-perovskite films (ITO/NiO_*x*_/PTAA/Al_2_O_3_-PMMA/WBG-perovskite-PMMA). **i** Space charge-limited current (SCLC) curves measured from forward (FW) and backward (BW) scans for hole-only devices (control: ITO/NiO_*x*_/PTAA/Al_2_O_3_/WBG-perovskite/PEAI/PTAA/Ag; DIC: ITO/NiO_*x*_/PTAA/Al_2_O_3_-PMMA/WBG-perovskite-PMMA/PEAI/PTAA/Ag). **j** Relationship between *V*_oc_ and light intensity for control and DIC-based solar cells

To elucidate the role of PMMA in WBG-perovskite crystal growth and film morphology, we investigated the crystallization kinetics and film formation processes (Figs. S12  and S13, Videos S1–S4). The crystallization time of the PMMA-incorporated DIC-perovskite film was significantly longer than that of the control films (i.e., WBG-perovskite), suggesting that PMMA modulates the nucleation and crystal growth processes. This prolonged crystallization process in the DIC-perovskite films integrating both BIC and GIC strategies promoted the formation of larger grains of 150–500 nm, whereas the control WBG-perovskite film displayed smaller grains of 50–350 nm (Fig. S14d–f). As further evidenced by the cross-sectional images (Figs. 3a, b and S14g), the DIC-perovskite film exhibits significantly improved morphological characteristics compared to the control film, displaying a nearly crack-free structure with enhanced compactness, high homogeneity, and well-defined perovskite crystals exhibiting preferential vertical growth without observable voids. This enhanced film uniformity can be attributed to the incorporation of PMMA as a polymeric Lewis base within the WBG-perovskite active layer, which likely serves as an effective crystal growth template [30, 45]. Thus, the more homogeneous morphology with larger grain sizes of the DIC-treated perovskite films enhances interfacial contacts and reduces shunting pathways, thereby contributing to increased *V*_oc_ and FF values in PIPVs.

Moreover, the hybrid PMMA/mp-Al_2_O_3_ ultrathin composite interlayer exhibits low-wetting properties (Fig. S2), which significantly enhance grain boundary mobility and promote the growth of larger grains. This phenomenon arises from the non-wetting substrate, which substantially mitigates surface tension-induced dragging forces. While hydrophobic surfaces of non-wetting films are beneficial for grain growth, they typically hinder the fabrication of continuous, pinhole-free perovskite films [54]. In contrast, our PMMA/mp-Al_2_O_3_ hybrid ultrathin composite interlayer with low-wetting property not only facilitates the uniform deposition of perovskite precursor but also supports controlled crystal growth. This conclusion was further supported by XRD pattern analysis, which revealed minimal residual PbI_2_ and highly oriented crystallization along the (110) plane in the DIC-perovskite film compared to control perovskite films (Fig. S15). Besides, the PMMA-incorporated DIC-perovskite films also showed an enhanced moisture-resisted properties (Fig. S16), significantly improving device stability through effective internal encapsulation by the hydrophobic polymer under ambient air conditions, as evidenced by Fig. 1e. Furthermore, the DIC-perovskite film showed improved surface morphology without obvious impurities and cracks (Fig. S14a–c). Photoluminescence (PL) mapping results as shown in Fig. S17 further confirm this conclusion. It can be observed that the control WBG-perovskite film showed PL heterogeneity, whereas the DIC-perovskite film showed a more uniform PL emission distribution (~ 725 nm).

To further study the surface electrical properties, we conducted conductive atomic force microscopy (C-AFM) and Kelvin probe force microscopy (KPFM) to analyze the current distribution and surface potential of both control- and DIC-perovskite films. As shown in Fig. 3c–f, while the control perovskite film exhibited obvious current and potential variations, the DIC-perovskite film demonstrated remarkable homogeneity in surface charge distribution. Both high potential fluctuation and current signal in the control perovskite film are caused by the more active surface defects, ion migration along GBs, and halide vacancies [55–57]. These factors collectively contribute to ionic conductivity and create leakage channels, which significantly deteriorate the *V*_oc_ and FF of photovoltaic devices under low-light conditions. Notably, the presence of PMMA and mp-Al_2_O_3_ significantly suppressed both surface current and potential variations, demonstrating its dual roles in passivating surface/interface defects and shunt paths.

In order to further investigate the charge transfer and extraction dynamics, we conducted steady-state PL and time-resolved PL spectra, photoluminescence quantum yield (PLQY), and space charge-limited current (SCLC) measurements on both control and DIC-based films and devices. Upon exciting from the bottom surface near the HTL, the DIC-based perovskite films incorporating HTLs showed an obvious PL quenching (Fig. S18a) and a fast decay lifetime compared to the control films (Fig. 3g), indicating efficient carrier extraction and the occurrence of a rapid interfacial hole transfer [58, 59]. The increase in PLQY from 1.21% to 4.05% (Figs. 3h and S18b) for the DIC-based WBG-perovskite corresponds to an enhancement in quasi-fermi level splitting (Δ*μ*) of ~ 30 meV. This gain directly contributes to the observed ~ 70 mV increased in *V*_oc_ (Fig. 1b and Table S2), further confirming efficient trap passivation. Further characterization of trap-state density for hole-only devices was analyzed by SCLC BW scans [60, 61], as depicted in Fig. 3i. The DIC-based hole-only device showed reduced hysteresis, a lower trap-filled limit voltage (*V*_TFL_) of 0.66 V and reduced trap density (*N*_t_) of 1.19 × 10^16^ cm^−3^ compared to the control device (*V*_TFL_ = 0.87 V, *N*_t_ = 1.52 × 10^16^ cm^−3^), indicating suppressed ion migration and nonradiative recombination. As shown in Fig. 3j, the ideality factor in the DIC-device decreased from 1.47 to 1.29, in good agreement with nonradiative recombination suppression and trap density reduction, and FF and *V*_oc_ enhancement. Dark *J-V* measurements and resistance analysis illustrated superior electrical properties in the DIC-based device, with a significantly higher shunt resistance (*R*_sh_ = 9.5 × 10^5^ Ω cm^2^) and lower current leakage loss compared to the control device (*R*_sh_ = 3.8 × 10^4^ Ω cm^2^), as shown in Fig. S19. Electrochemical impedance spectroscopy (EIS) analysis through Nyquist plot fitting (Fig. S20) provided additional insights into the carrier transport resistance and recombination kinetics. Both devices showed two characteristic semicircles corresponding to charge recombination resistance (*R*_1_) and dielectric relaxation resistance (*R*_2_). The DIC-based device demonstrated remarkably higher resistance values (*R*_1_ = 4413 Ω, *R*_2_ = 1424 Ω) compared to the control device (*R*_1_ = 2496 Ω and *R*_2_ = 656.5 Ω) (Table S10), which directly correlates with improved FF and *V*_oc_ characteristics [62, 63]. These improved properties explain the superior *V*_oc_ and FF values observed in DIC-devices under low-light conditions (Fig. 2d and Table 1).

### Passivation Mechanism of Dual Insulator Contacts in WBG-Perovskite Films

**Fig. 4:**
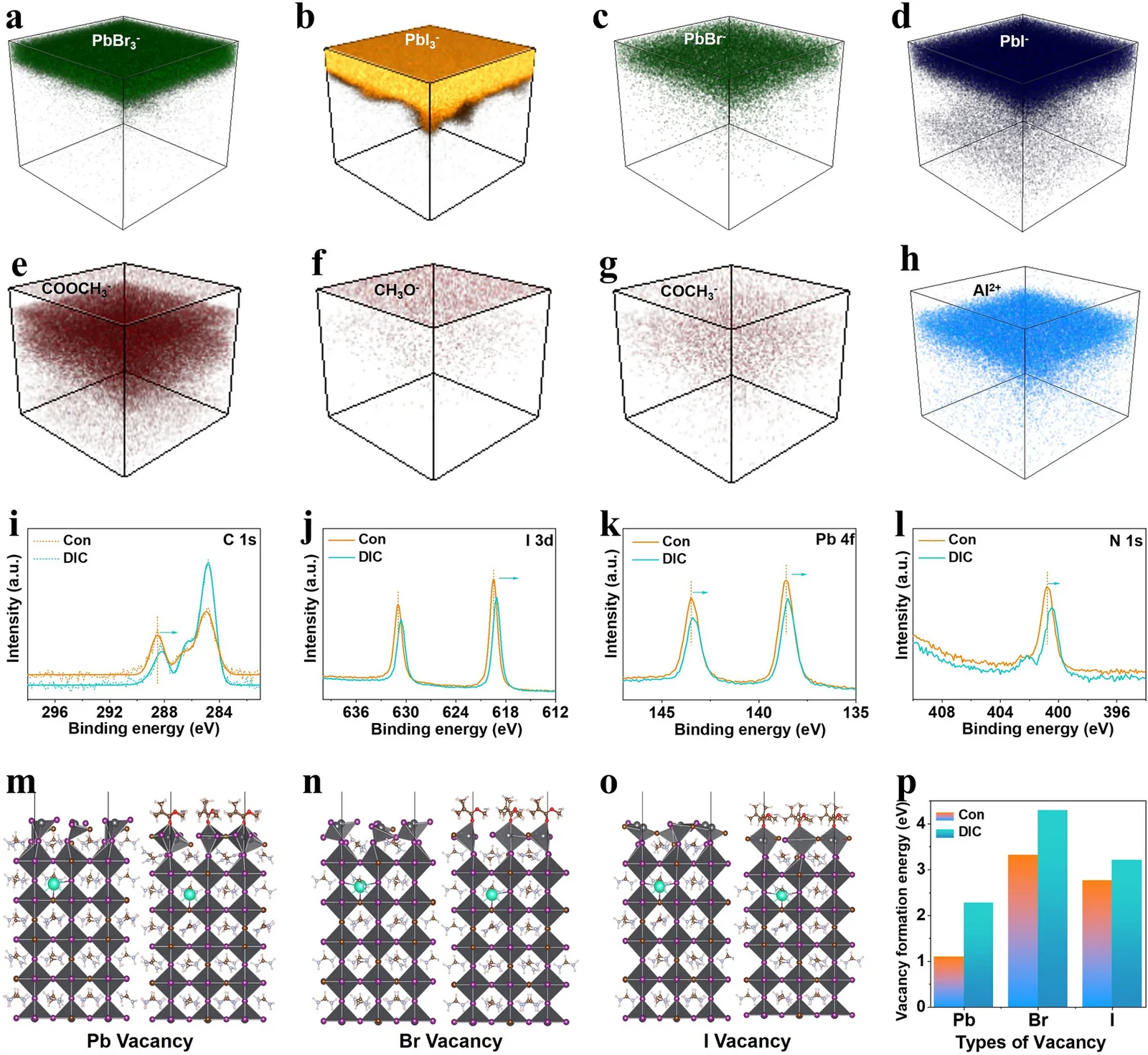
Passivation mechanism of dual insulator contacts (DIC) in WBG-perovskite films. ToF–SIMS 3D tomography images of elemental distribution for DIC-perovskite film of ITO/NiO_*x*_/PTAA/mp-Al_2_O_3_-PMMA/WBG-perovskite-PMMA: **a** ${PbBr}_{3}^{-}$, **b** ${PbI}_{3}^{-}$, **c** ${PbBr}^{-}$, **d** ${PbI}^{-}$, **e** ${COOCH}_{3}^{-}$, **f** ${{CH}_{3}O}^{-}$, **g** ${COCH}_{3}^{-}$ and **h** ${Al}^{2+}$. XPS spectra of **i** C 1 *s*, **j** I 3*d*, **k** Pb 4*f* and **l** N 1 *s*. DFT calculations of **m** Pb, **n** Br and **o** I vacancies. **p** Formation energies of antisites and Schottky vacancies

Figure S21 presents time-of-flight secondary ion mass spectrometry (ToF–SIMS) depth profile for DIC-based film with a configuration of ITO/HTL/PMMA-Al_2_O_3_/PMMA-PVK, showing the negative ion distribution of ${CO}^{-}{, COOCH}_{3}^{-}$, ${{CH}_{3}O}^{-},$ and ${COCH}_{3}^{-}$ from PMMA in the entire WBG-perovskite bulk heterostructures and hybrid ultrathin composite interlayer. The ToF–SIMS 3D tomography as shown in Fig. 4a–h further distinctly reveals the distribution of these negative ions and Al^2+^ within the WBG-perovskites and at the buried interface, respectively. These findings suggest the presence of PMMA and mp-Al_2_O_3_-PMMA in the WBG-perovskite bulk heterostructures and at its buried interface, respectively.

To further explore the passivation mechanism of polymer PMMA in the WBG-perovskite bulk heterostructures and hybrid ultrathin composite interlayer, we conducted X-ray photoelectron spectroscopy (XPS) spectra of the DIC-based perovskite film. Compared to the control WBG-perovskite film, the peak of C 1*s*, I 3*d*, Pb 4*f* and N 1*s* in DIC-based WBG-perovskite film shifted toward lower binding energy by ~ 0.3 V from the Fermi level (*E*_f_) (Fig. 4i–l), indicating the presence of chemical bonding and interaction between PMMA and WBG-perovskite molecules, and ~ 0.3 eV upward shift of the valence band maximum [64]. This is likely associated with the carbonyl groups (C=O) in PMMA acting as Lewis base site, donating its lone pair of electrons to the uncoordinated Pb ions [29, 30, 65, 66], eliminating the corresponding trap and reducing undesired nonradiative recombination. To confirm this, we also calculated formation energies of antisites and Schottky vacancies including Pb, I, and Br by density functional theory (DFT) simulations. Our DFT calculations reveal that the DIC strategy significantly increases formation energies for key defects (Pb/I/Br antisites and Schottky vacancies) in WBG-perovskites (Fig. 4m–p). This thermodynamic stabilization likely originates from two synergistic mechanisms: (i) Coordinative bonding at grain boundaries. The carbonyl (C=O) groups in PMMA form strong Lewis acid–base interactions with undercoordinated Pb^2+^ at GBs. (ii) Creates electrostatic screening that destabilizes antisite defects and modulates local electrostatic potential. In the view of electronic structure implications, the higher defect formation energies correlate with reduced mid-gap states in density of states and increased charge carrier lifetimes as discussed above, which indicates the excellent passivation of PMMA by coordinative bonding with the defect sites at GBs and/or perovskite/HTL heterointerface, effectively suppressing nonradiative recombination. This multi-scale passivation from atomic-scale coordinative bonding to mesoscale defect energy modulation clearly explains the exceptional *V*_oc_ and FF improvements in DIC devices.

## Conclusions

In conclusion, we developed a dual insulator contact (DIC) strategy that integrates both grain boundary insulator contact (GIC) and buried interface insulator contact (BIC) architectures to effectively mitigate defect-induced nonradiative recombination in WBG-perovskite films. The GIC structure, utilizing PMMA polymer, effectively passivates grain boundary and bulk defects through carbonyl groups (C=O) coordination with uncoordinated Pb^2+^ ions, while the BIC architecture employs a PMMA/mp-Al_2_O_3_ hybrid ultrathin composite interlayer to address interface defects. The GIC architecture enables the formation of bulk heterojunction structures, effectively passivating defects at GBs and/or voids within WBG-perovskite films. The BIC structure primarily addresses interface defects through the incorporation of a hybrid ultrathin composite interlayer featuring homogeneous distribution of mp-Al_2_O_3_ and PMMA polymer at the HTL/WBG-perovskite interface. This synergistic approach yields WBG-PIPVs with improved indoor efficiencies across multiple illumination intensities: 44.36% (*V*_oc_ of 1.091 V and FF of 83.97%) at 1,000 lx (288.4 µW cm^−2^ and 2950 K) to 40.94% (*V*_oc_ of 1.020 V and FF of 82.83%) at 200 lx (57.77 µW cm^−2^ and 2950 K) under LED lighting. Our findings demonstrate that precisely engineered insulator contacts can simultaneously suppress shunting pathways, reduce vacancy formation, and minimize recombination losses. This DIC strategy provides an effective strategy for developing high-performance IPVs to power next-generation IoT electronics in low-light environments.

## Supplementary Information

Below is the link to the electronic supplementary material.

Supplementary file1 (DOCX 26086 KB)

Supplementary file2 (MP4 2856 KB)

Supplementary file3 (MP4 3040 KB)

Supplementary file4 (MP4 3200 KB)

Supplementary file5 (MP4 3555 KB)
