# Supplementary material for: Electronic Blockade of Shunting Pathways via Dual Insulator Contacts for High-Efficiency Wide-Bandgap Perovskite Indoor Photovoltaics
Source: Nanomicro Lett. 2026 May 19;18:375. doi: 10.1007/s40820-026-02225-5 (PMC13187103; doi:10.1007/s40820-026-02225-5)
Supplement: Supplementary file 1 — Supplementary file1 (DOCX 26086 KB) [file 40820_2026_2225_MOESM1_ESM.docx]

Supporting Information for

**Electronic Blockade of Shunting Pathways *via* Dual Insulator Contacts for High-Efficiency Wide-Bandgap Perovskite Indoor Photovoltaics**

Quanxi Liu^1, 2^, Yousheng Wang^1,^ * Qiaoyan Ma^1^, Jianzha Zheng^1, 4^, Yinghui Peng^1^, Liwei Wang^1^, Zeyu Chen^1^, Tianhao Du^1^, Daxin Xiao^1^, Jiandong Fan^1,^ *, Yoon-Bong Hahn^5^ and Yaohua Mai^1, 3,^ *

^1^ Institute of New Energy Technology, College of Physics & Optoelectronic Engineering, Jinan University, Guangzhou 510632, P. R. China

^2^ Department of Electronic Engineering, College of Information Science and Technology, Jinan University, Guangzhou 510632, P. R. China

^3^ Guangdong Mellow Energy Co., Ltd, Zhuhai 519000, P. R. China

^4^ Institute of Applied Physics and Materials Engineering, University of Macau, Macao 999078, P. R. China

^5^ School of Semiconductor and Chemical Engineering, Solar Energy Research Center, Jeonbuk National University, 567 Baekjedaero, Deokjin-gu, Jeonju-si, Jeollabuk-do, 54896 Republic of Korea

*Corresponding authors. E-mail: [wangys0120@jnu.edu.cn](mailto:wangys0120@jnu.edu.cn) (Yousheng Wang); [jdfan@jnu.edu.cn](mailto:jdfan@jnu.edu.cn) (Jiandong Fan); [yaohuamai@jnu.edu.cn](mailto:yaohuamai@jnu.edu.cn) (Yaohua Mai)

**Supplementary Figures and Tables**

**Fig. S1** Current density-voltage (*J-V*) characteristics obtained at two working distances (*WD*_A_ = 37.5 cm and *WD*_B_ = 18.4 cm) from light-emitting diode (1,000 lx, 288.4 µW cm^-2^, 2,950 K)

**Table S1 Photovoltaic parameters of WBG-PIPVs under light-emitting diode (1,000 lx, 288.4 µW cm^-2^, 2,950 K) illumination at two working distances (*WD*_A_ = 37.5 cm and *WD*_B_ = 18.4 cm).** The angular effects were assessed by comparing indoor performance at two different distances of 37.5 cm (A) and 18.4 cm (B) under the same illuminance (1,000 lx), which confirmed a deviation (ε) of less than 5% (ε = $\frac{\text{PCE}_{B}\text{-}\text{PCE}_{A}}{\text{PCE}_{B}}$= (42.72-41.73)/42.72≤2.32%).

| **Distance** | **Scan direction** | **PCE(i) (%)** | ***V*_oc_ (V)** | ***J*_sc_ (μA/cm^2^)** | **FF (%)** | ***P*_out_ (μW cm^-2^)** |
| --- | --- | --- | --- | --- | --- | --- |
| 18.4 cm | RS | 42.72 | 1.0745 | 138.37 | 82.86 | 123.20 |
|  | FS | 41.93 | 1.061 | 138.34 | 82.39 | 120.93 |
| 37.5 cm | RS | 41.73 | 1.0745 | 136.44 | 82.10 | 120.37 |
|  | FS | 41.00 | 1.061 | 136.52 | 81.63 | 118.24 |


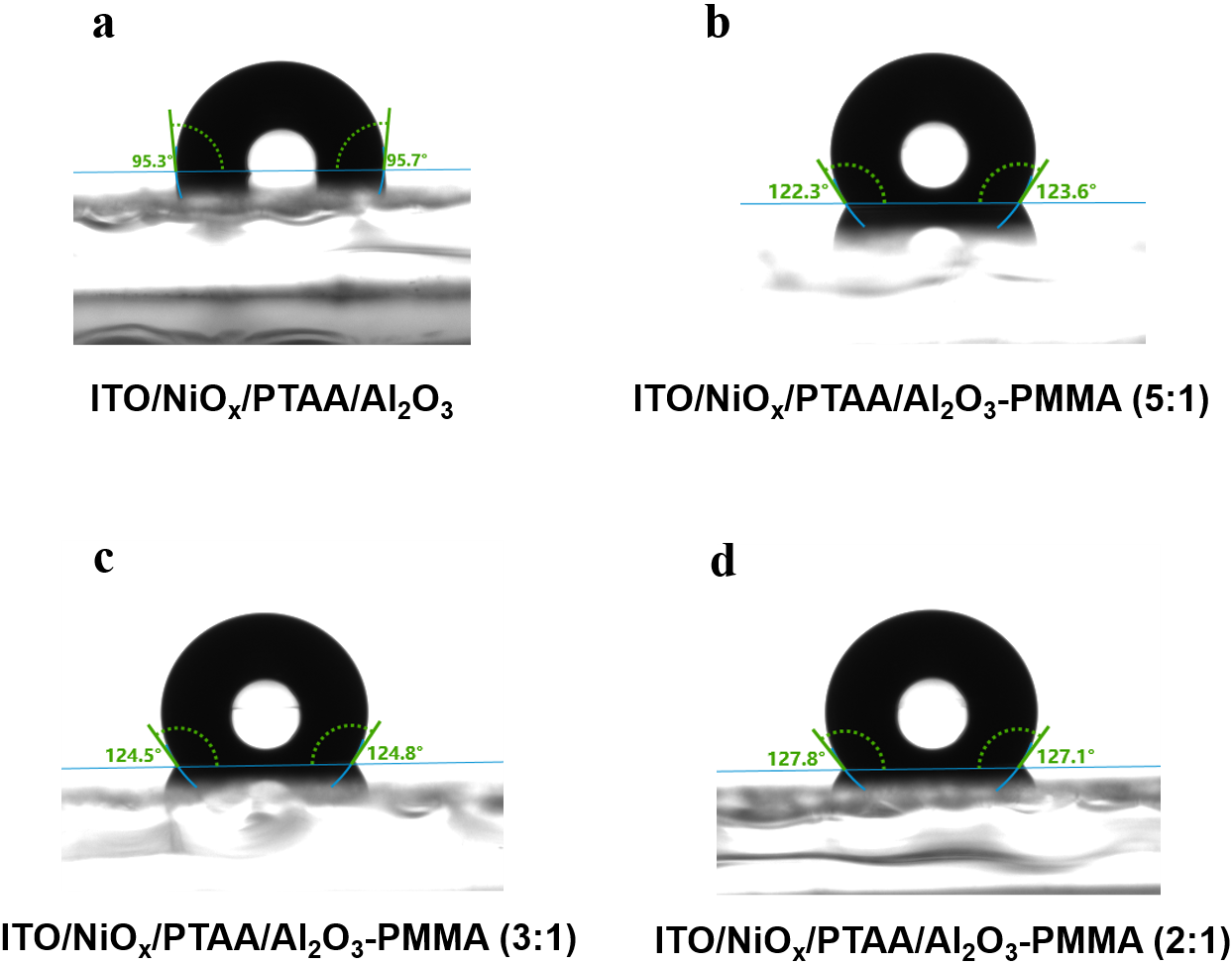


**Fig. S2** **Contact angles of the perovskite precursor on a hybrid ultrathin layer with different contents of PMMA:** **a** without PMMA, **b-d** ratio of mp-Al_2_O_3_ to PMMA of 5:1, 3:1 and 2:1

**
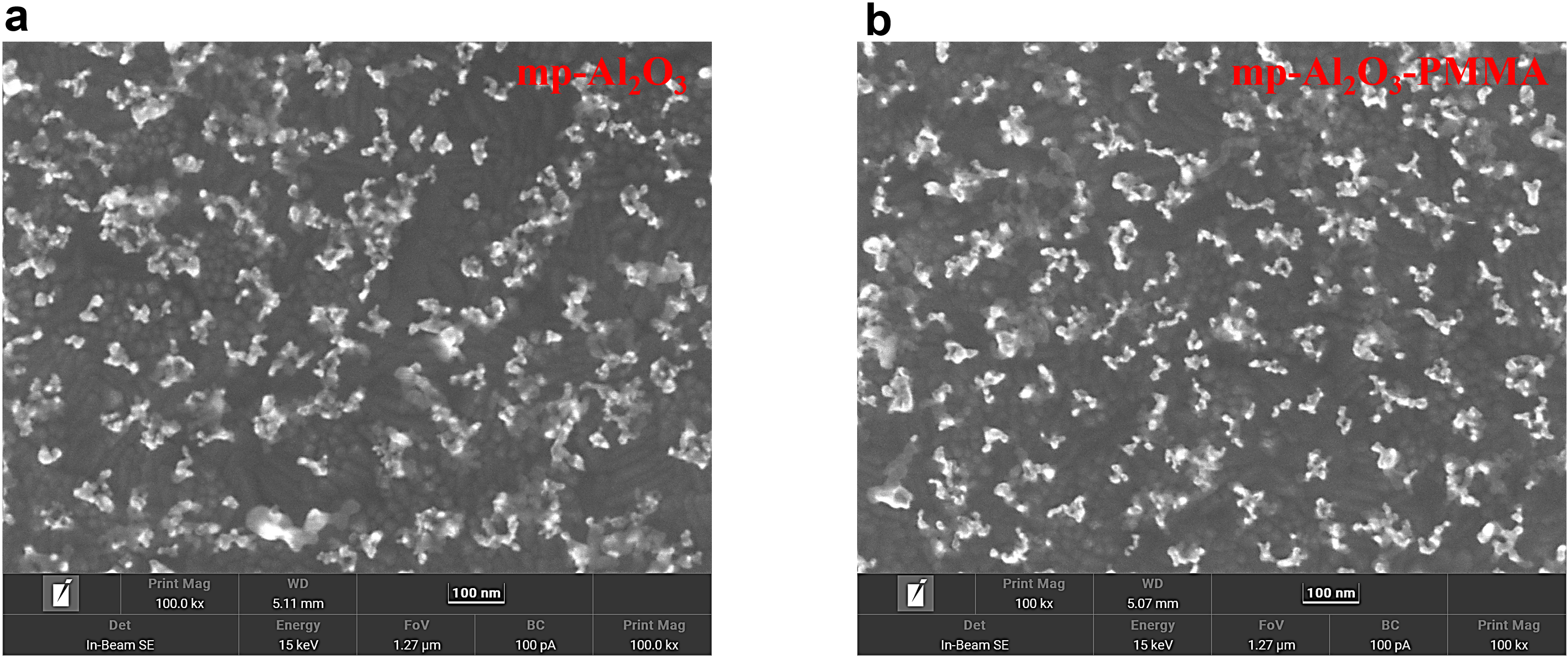
**

**Fig. S3** **SEM surface images of mp-Al_2_O_3_-PMMA hybrid ultrathin layer:** **a** mp-Al_2_O_3_ and **b** mp-Al_2_O_3_-PMMA

**Table S2** Photovoltaic parameters of WBG-PIPVs under light-emitting diode (1,000 lx, 288.4 µW cm^-2^, 2,950 K) illumination for the optimal four types of cells

| **Devices** | **Scan direction** | **PCE(i) (%)** | ***V*_oc_ (V)** | ***J*_sc_ (μA/cm^2^)** | **FF (%)** | ***P*_out_ (μW cm^-2^)** |
| --- | --- | --- | --- | --- | --- | --- |
| Con | Rs | 39.06 | 1.018 | 137.11 | 80.70 | 112.64 |
|  | Fs | 36.34 | 1.005 | 136.99 | 76.12 | 104.80 |
| GIC | Rs | 42.02 | 1.064 | 137.43 | 82.88 | 121.19 |
|  | Fs | 41.33 | 1.057 | 137.36 | 82.09 | 119.19 |
| BIC | Rs | 41.75 | 1.028 | 138.00 | 84.87 | 120.40 |
|  | Fs | 40.66 | 1.016 | 138.01 | 83.63 | 117.27 |
| DIC | Rs | 44.36 | 1.091 | 138.72 | 83.97 | 127.94 |
|  | Fs | 43.51 | 1.082 | 137.82 | 83.37 | 125.47 |

**
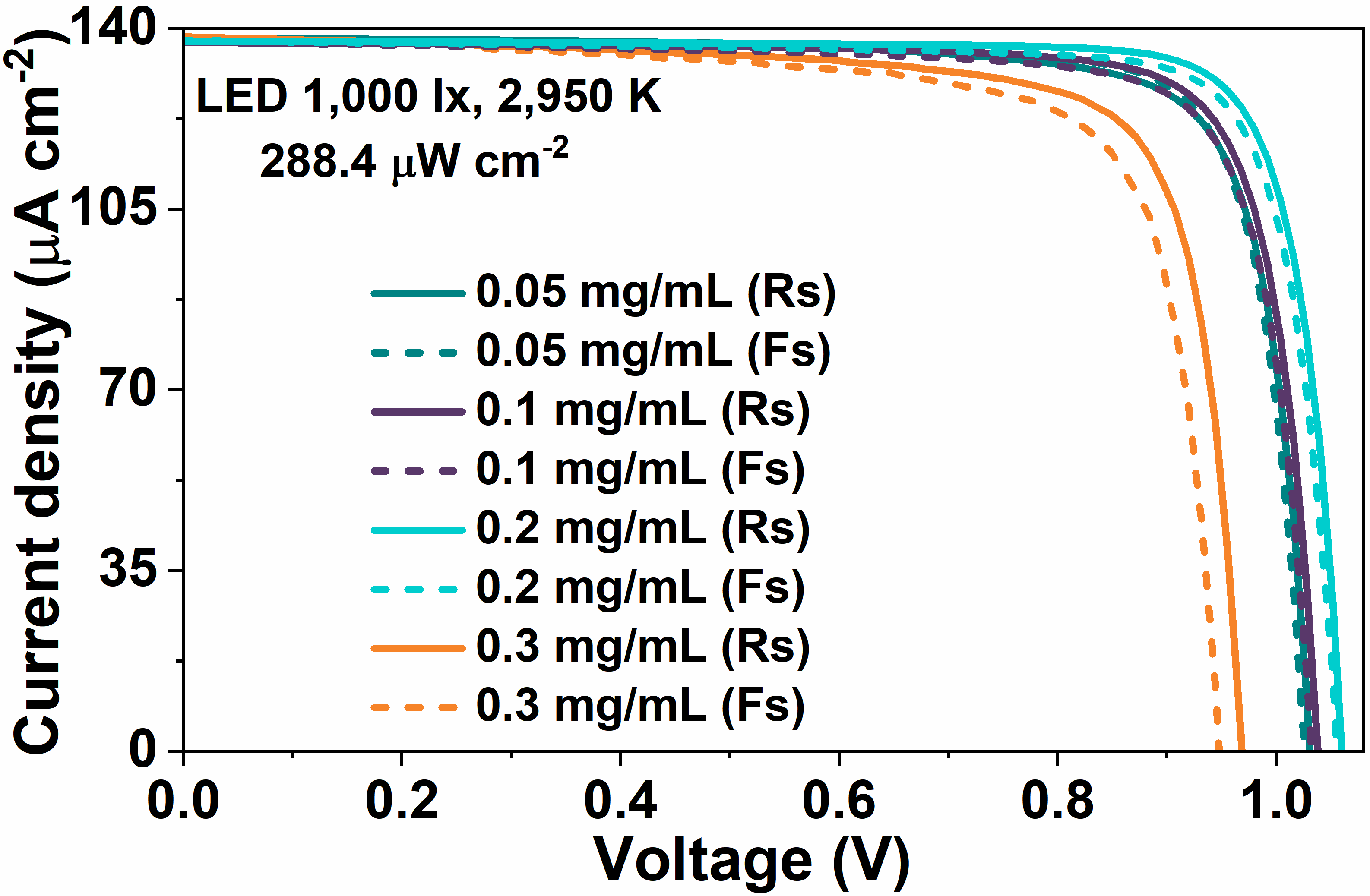
**

**Fig. S4** *J-V* performance of both reverse and forward scan under LED illumination (1,000 lx, 288.4 µW cm^-2^, 2,950 K) for different contents of PMMA in EA antisolvent

**Table S3** Photovoltaic parameters of WBG-PIPVs under light-emitting diode (1,000 lx, 288.4 µW cm^-2^, 2,950 K) illumination for different contents of PMMA in EA antisolvent

| **Contents of PMMA** | **Scan direction** | **PCE(i) (%)** | ***V*_oc_ (V)** | ***J*_sc_ (μA/cm^2^)** | **FF (%)** | ***P*_out_ (μW cm^-2^)** |
| --- | --- | --- | --- | --- | --- | --- |
| 0.05 | Rs | 40.27 | 1.028 | 138.11 | 81.80 | 116.14 |
|  | Fs | 39.81 | 1.028 | 138.04 | 80.89 | 114.79 |
| 0.1 | Rs | 40.63 | 1.040 | 137.34 | 82.03 | 117.17 |
|  | Fs | 39.83 | 1.028 | 137.56 | 81.22 | 114.86 |
| 0.2 | Rs | 42.60 | 1.064 | 137.56 | 83.93 | 122.85 |
|  | Fs | 41.80 | 1.052 | 137.72 | 83.20 | 120.55 |
| 0.3 | Rs | 36.28 | 0.968 | 138.38 | 78.11 | 104.63 |
|  | Fs | 34.58 | 0.944 | 138.60 | 76.22 | 99.73 |

**Fig. S5** Performance statistics of reverse and forward scan in WBG-PIPVs (15~20 cells) measured under LED illumination (1,000 lx, 288.4 µW cm^-2^, 2950 K) for different contents of PMMA in EA solvent: **a** *i*-PCE, **b** *V*_oc_ and **c** FF

**
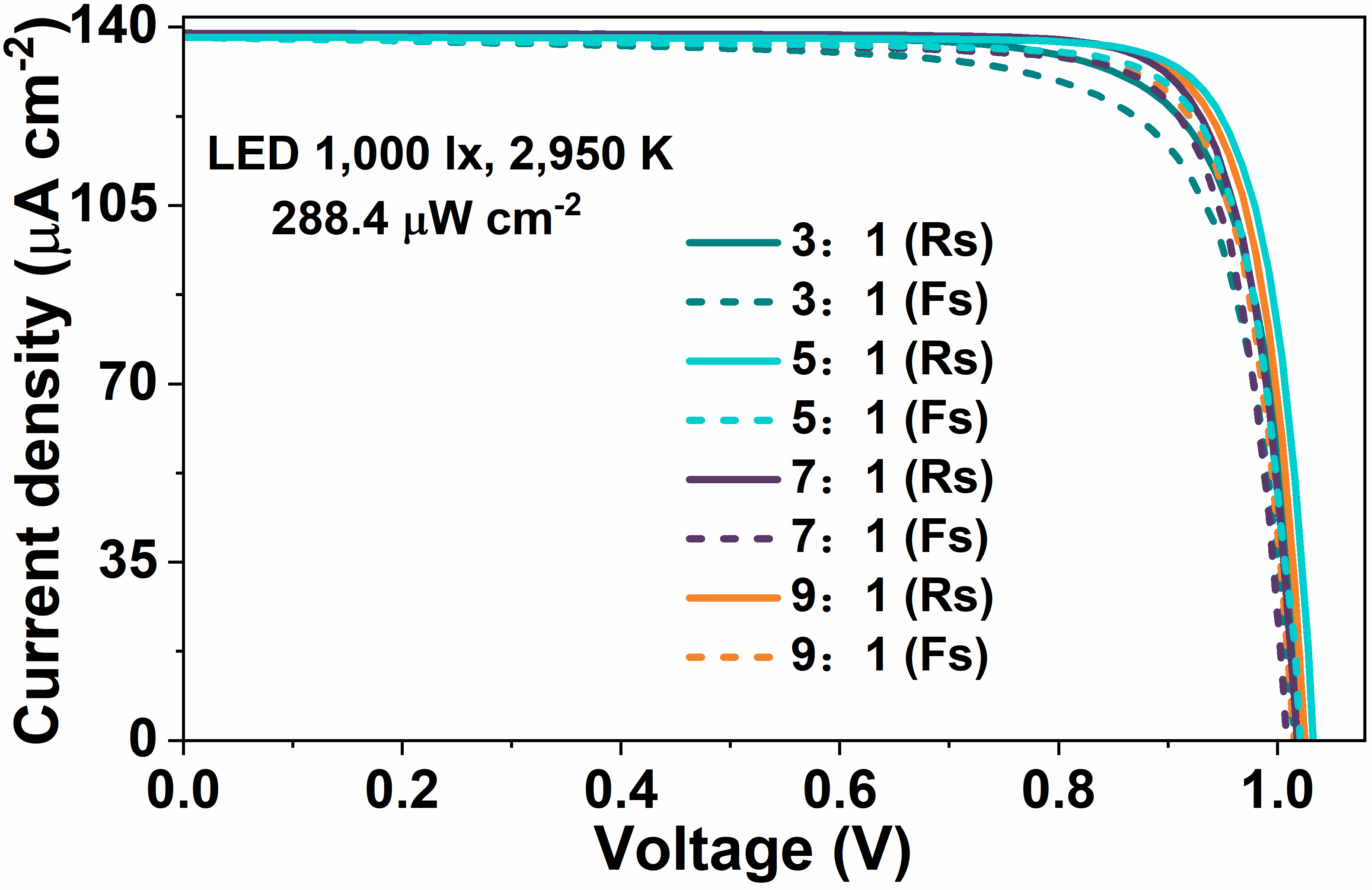
**

**Fig. S6** *J-V* performance of both reverse and forward scan under LED illumination (1,000 lx, 288.4 µW cm^-2^, 2,950 K) for different ratios of mp-Al_2_O_3_ to PMMA as a hybrid ultrathin layer

**Table S4** Photovoltaic parameters of WBG-PIPVs under light-emitting diode (1,000 lx, 288.4 µW cm^-2^, 2,950 K) illumination for different ratios of mp-Al_2_O_3_ to PMMA as a hybrid ultrathin layer

| **Ratio of mp-Al_2_O_3_ to PMMA** | **Scan direction** | **PCE(i) (%)** | ***V*_oc_ (V)** | ***J*_sc_ (μA/cm^2^)** | **FF (%)** | ***P*_out_ (μW cm^-2^)** |
| --- | --- | --- | --- | --- | --- | --- |
| 3：1 | Rs | 39.04 | 1.028 | 138.08 | 79.32 | 112.60 |
|  | Fs | 36.93 | 1.016 | 138.22 | 75.84 | 106.51 |
| 5：1 | Rs | 41.75 | 1.028 | 138.00 | 84.87 | 120.40 |
|  | Fs | 40.67 | 1.016 | 138.03 | 83.63 | 117.28 |
| 7：1 | Rs | 40.82 | 1.016 | 138.78 | 83.49 | 117.77 |
|  | Fs | 39.37 | 1.004 | 138.96 | 81.39 | 113.56 |
| 9：1 | Rs | 41.15 | 1.028 | 138.54 | 83.32 | 118.67 |
|  | Fs | 39.73 | 1.016 | 138.66 | 81.31 | 114.56 |

**Fig. S7** Performance statistics of reverse and forward scan in WBG-PIPVs (15~20 cells) measured under LED illumination (1,000 lx, 288.4 µW cm^-2^, 2,950 K) for different ratios of mp-Al_2_O_3_ to PMMA as a hybrid ultrathin layer: a *i*-PCE, b *V*_oc_ and c FF

**Table S5** Summarized photovoltaic parameters of control devices under light-emitting diode (1,000 lx, 288.4 µW cm^-2^, 2,950 K) illumination

| **#** | **PCE(i) (%)** | ***V*_oc_ (V)** | ***J*_sc_ (μA/cm^2^)** | **FF (%)** | ***P*_out_ (μW cm^-2^)** |
| --- | --- | --- | --- | --- | --- |
| 1 | 36.63 | 1.010 | 135.22 | 77.34 | 105.62 |
| 2 | 36.88 | 1.018 | 132.54 | 78.83 | 106.36 |
| 3 | 37.86 | 1.010 | 138.48 | 78.07 | 109.19 |
| 4 | 38.51 | 1.012 | 135.71 | 80.85 | 111.04 |
| 5 | 38.17 | 1.001 | 137.17 | 80.16 | 110.07 |
| 6 | 36.67 | 1.018 | 136.09 | 76.33 | 105.77 |
| 7 | 38.05 | 1.005 | 136.50 | 80.00 | 109.74 |
| 8 | 37.86 | 1.004 | 137.04 | 79.36 | 109.19 |
| 9 | 34.55 | 0.944 | 137.07 | 77.00 | 99.64 |
| 10 | 36.19 | 0.980 | 134.80 | 79.00 | 104.36 |
| 11 | 36.91 | 0.956 | 137.49 | 80.97 | 106.43 |
| 12 | 38.58 | 1.016 | 135.23 | 80.97 | 111.25 |
| 13 | 35.74 | 0.992 | 131.50 | 79.00 | 103.06 |
| 14 | 39.06 | 1.016 | 136.76 | 81.07 | 112.65 |
| 15 | 38.28 | 1.018 | 135.24 | 80.20 | 110.41 |
| Ave. | 37.33 | 1.00 | 135.78 | 79.28 | 107.65 |

**Table S6** Summarized photovoltaic parameters of GIC-based WBG-PIPVs under light-emitting diode (1,000 lx, 288.4 µW cm^-2^, 2,950 K) illumination

| **#** | **PCE(i) (%)** | ***V*_oc_ (V)** | ***J*_sc_ (μA/cm^2^)** | **FF (%)** | ***P*_out_ (μW cm^-2^)** |
| --- | --- | --- | --- | --- | --- |
| 1 | 40.48 | 1.028 | 138.29 | 82.12 | 116.74 |
| 2 | 38.83 | 1.035 | 134.32 | 80.54 | 111.97 |
| 3 | 38.69 | 1.018 | 134.33 | 81.59 | 111.57 |
| 4 | 37.91 | 1.015 | 134.62 | 80.01 | 109.33 |
| 5 | 39.32 | 1.055 | 133.70 | 80.38 | 113.38 |
| 6 | 37.57 | 1.015 | 131.81 | 80.99 | 108.35 |
| 7 | 38.14 | 1.018 | 133.97 | 80.65 | 109.99 |
| 8 | 40.77 | 1.028 | 138.40 | 82.64 | 117.57 |
| 9 | 42.03 | 1.064 | 137.43 | 82.88 | 121.19 |
| 10 | 39.89 | 1.031 | 138.73 | 80.44 | 115.05 |
| 11 | 40.55 | 1.028 | 139.67 | 81.44 | 116.93 |
| 12 | 39.33 | 1.018 | 137.54 | 81.01 | 113.42 |
| 13 | 40.09 | 1.045 | 140.01 | 79.01 | 115.62 |
| 14 | 41.26 | 1.055 | 136.16 | 82.84 | 118.99 |
| 15 | 40.41 | 1.040 | 138.57 | 80.86 | 116.53 |
| Ave. | 39.71 | 1.033 | 136.57 | 81.22 | 114.50 |

**Table S7** Summarized photovoltaic parameters of BIC-based WBG-PIPVs under light-emitting diode (1,000 lx, 288.4 µW cm^-2^, 2,950 K) illumination

| **#** | **PCE(i) (%)** | ***V*_oc_ (V)** | ***J*_sc_ (μA/cm^2^)** | **FF (%)** | ***P*_out_ (μW cm^-2^)** |
| --- | --- | --- | --- | --- | --- |
| 1 | 39.75 | 0.980 | 138.31 | 84.57 | 114.63 |
| 2 | 40.15 | 1.016 | 137.93 | 82.63 | 115.79 |
| 3 | 41.05 | 1.016 | 138.92 | 83.88 | 118.38 |
| 4 | 40.22 | 1.016 | 138.23 | 82.00 | 116.00 |
| 5 | 39.47 | 1.004 | 135.04 | 83.96 | 113.84 |
| 6 | 41.22 | 1.014 | 139.54 | 84.02 | 118.88 |
| 7 | 39.51 | 1.018 | 132.72 | 84.32 | 113.93 |
| 8 | 41.06 | 1.018 | 137.76 | 84.43 | 118.41 |
| 9 | 39.19 | 1.016 | 135.96 | 81.82 | 113.02 |
| 10 | 40.29 | 1.020 | 136.12 | 83.68 | 116.18 |
| 11 | 40.03 | 1.016 | 134.74 | 84.34 | 115.46 |
| 12 | 40.81 | 1.004 | 138.66 | 84.54 | 117.70 |
| 13 | 40.62 | 1.024 | 136.81 | 83.62 | 117.15 |
| 14 | 41.36 | 1.016 | 138.73 | 84.62 | 119.27 |
| 15 | 39.43 | 0.992 | 138.44 | 82.79 | 113.70 |
| 16 | 41.75 | 1.028 | 138.00 | 84.87 | 120.40 |
| 17 | 39.51 | 0.992 | 138.58 | 82.28 | 113.93 |
| 18 | 40.66 | 1.004 | 138.30 | 84.45 | 117.27 |
| 19 | 40.28 | 1.016 | 139.41 | 82.02 | 116.17 |
| 20 | 40.93 | 1.016 | 138.83 | 83.71 | 118.08 |
| Ave. | 40.45 | 1.011 | 137.57 | 83.63 | 116.64 |

**Table S8** Summarized photovoltaic parameters of DIC-based WBG-PIPVs under light-emitting diode (1,000 lx, 288.4 µW cm^-2^, 2,950 K) illumination

| **#** | **PCE(i) (%)** | ***V*_oc_ (V)** | ***J*_sc_ (μA/cm^2^)** | **FF (%)** | ***P*_out_ (μW cm^-2^)** |
| --- | --- | --- | --- | --- | --- |
| 1 | 43.48 | 1.064 | 139.48 | 84.50 | 125.40 |
| 2 | 42.64 | 1.057 | 137.82 | 84.41 | 122.96 |
| 3 | 41.48 | 1.057 | 136.42 | 82.96 | 119.63 |
| 4 | 42.46 | 1.057 | 137.79 | 84.07 | 122.44 |
| 5 | 41.27 | 1.057 | 136.87 | 82.28 | 119.03 |
| 6 | 42.42 | 1.070 | 136.74 | 83.62 | 122.34 |
| 7 | 41.27 | 1.057 | 135.30 | 83.22 | 119.01 |
| 8 | 41.99 | 1.070 | 135.57 | 83.48 | 121.10 |
| 9 | 43.10 | 1.083 | 138.09 | 83.11 | 124.29 |
| 10 | 43.49 | 1.083 | 138.43 | 83.66 | 125.42 |
| 11 | 42.93 | 1.076 | 137.30 | 83.79 | 123.79 |
| 12 | 41.02 | 1.057 | 136.07 | 82.24 | 118.28 |
| 13 | 43.12 | 1.070 | 139.06 | 83.57 | 124.35 |
| 14 | 44.36 | 1.091 | 138.72 | 83.97 | 127.94 |
| 15 | 43.51 | 1.082 | 137.82 | 83.37 | 125.47 |
| 16 | 43.71 | 1.083 | 138.17 | 84.24 | 126.06 |
| 17 | 43.74 | 1.083 | 138.72 | 83.96 | 126.14 |
| 18 | 42.58 | 1.070 | 137.82 | 83.27 | 122.80 |
| 19 | 43.59 | 1.083 | 137.69 | 84.30 | 125.71 |
| 20 | 43.15 | 1.076 | 138.09 | 83.75 | 124.44 |
| Ave. | 42.93 | 1.068 | 137.57 | 83.83 | 123.81 |





**Fig. S8** Steady-state power output (SPO) curves of the champion DIC device at the maximum power point (MPP) *V*_oc_ of 0.944 V under LED illumination (1,000 lx, 288.4 µW/cm^2^, 2,950 K) for 1200 s

**Fig. S9** EQE spectra and the integrated current density for the control and DIC champion devices under 1,000 lx illumination

**
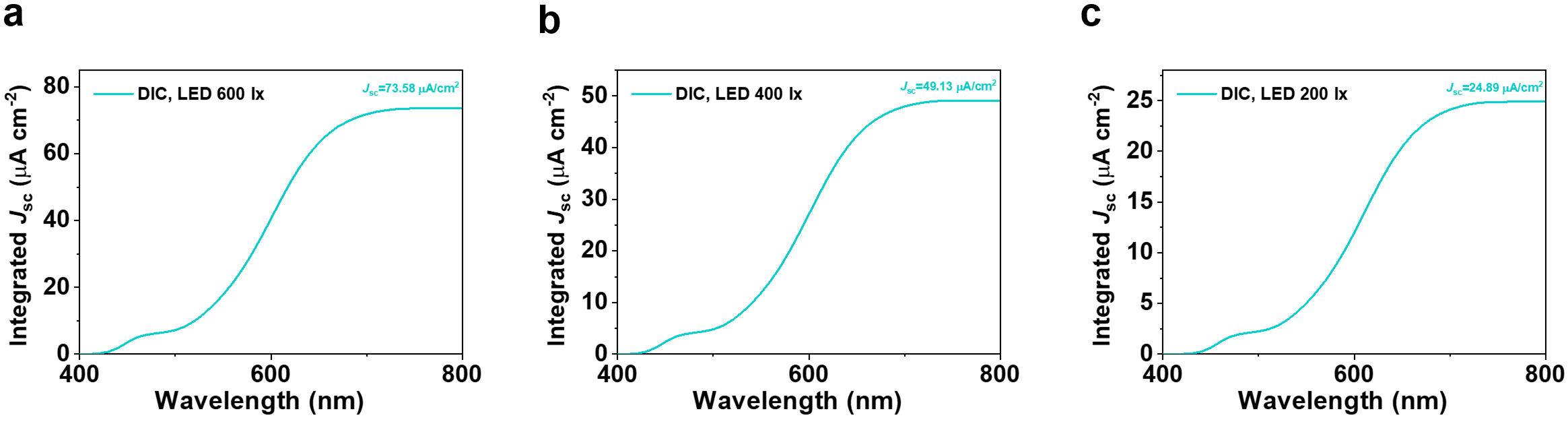
**

**Fig. S10** Integrated current density from EQE spectra of DIC champion devices under (**a**) 600 lx, (**b**) 400 lx and (**c**) 200 lx illumination, respectively

**
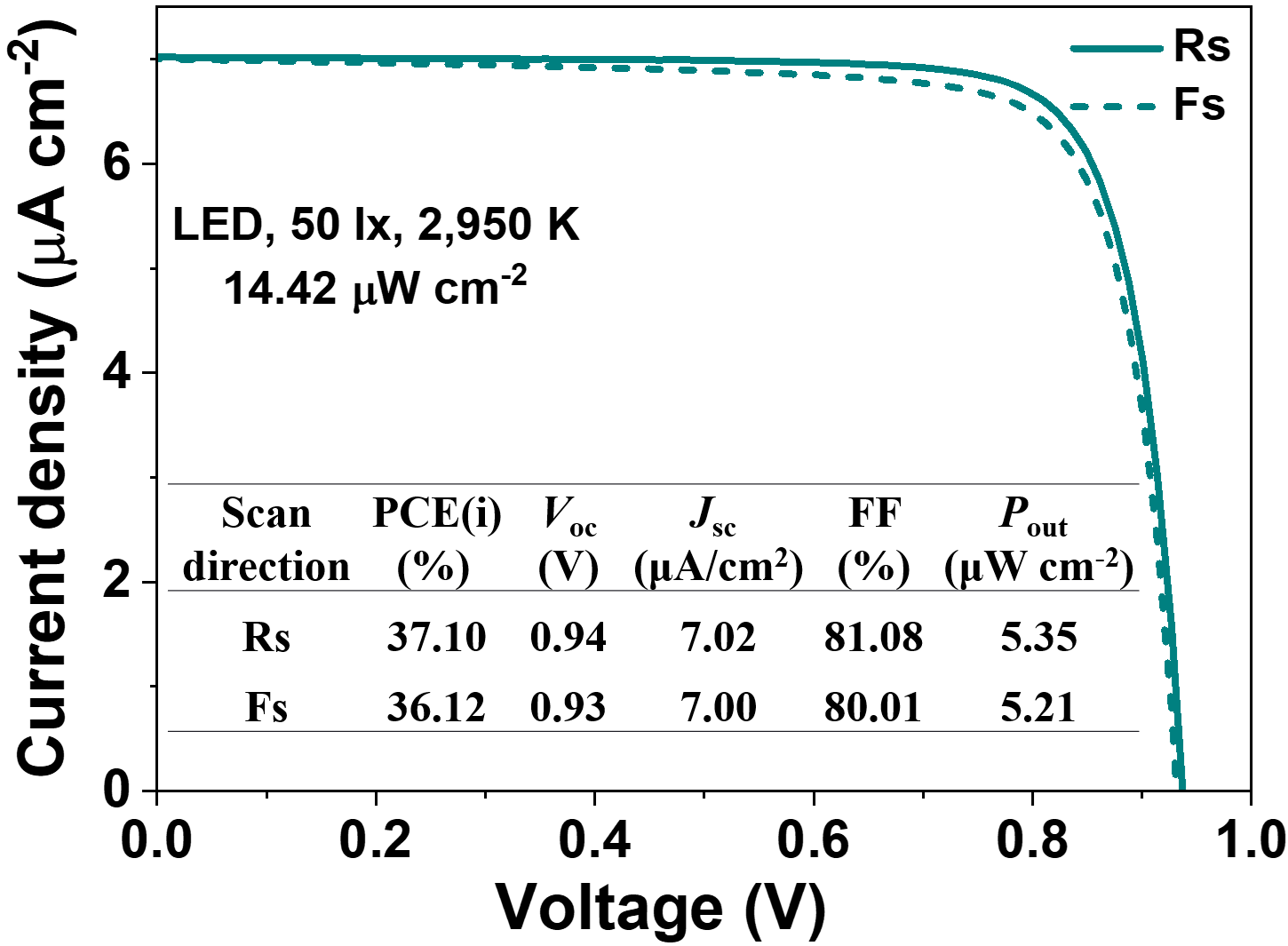
**

**Fig. S11** *J-V* curves and corresponding photovoltaic parameters incorporated reverse and forward scans of the DIC-device under 50 lx illumination

**Table S9** Reported efficiencies of PIPVs under low irradiances (~600 lx) illumination conditions (LED)

| Perovskite  compositions | E_g_  (eV) | Illumination conditions | Active/aperture area (cm^2^) | *V_oc_* (V) | FF  (%) | PCE(i)  (%) | *P*_out_  (μW cm^-2^) | Refs. |
| --- | --- | --- | --- | --- | --- | --- | --- | --- |
| (FAPbI_3_)_0.97_(MAPbBr_3_)_0.03_ | 1.59 | 500 lx, 170 μW cm^-2^,  2,700 K | 0.08 | 0.92 | 79.01 | 35.55 | N/A | [S1] |
|  |  | 200 lx, 71 μW cm^-2^,  2,700 K  (DYSON, CD05) |  | 0.89 | 72.44 | 34.77 | N/A |  |
| FAPbI_3_ | 1.52 | 531 lx, 167 μW cm^-2^,  2,956 K | 0.07 | 0.89 | 79.7 | 30.56 | 51.0 | [S2] |
|  |  | 212 lx, 83 μW cm^-2^,  2,956 K |  | 0.84 | 80.3 | 30.65 | 20.5 |  |
|  |  | 106 lx, 42 μW cm^-2^,  2,956 K  (N/A) |  | 0.83 | 80.7 | 29.89 | 9.98 |  |
| Cs_0.17_FA_0.83_PbI_1.8_Br_1.2_ | 1.77 | 600 lx, 181 μW cm^-2^,  3,000 K | 0.09 | 1.01 | 80.46 | 32.93 | 59.66 | [S3] |
|  |  | 400 lx, 121 μW cm^-2^,  3,000 K |  | 0.99 | 79.08 | 31.96 | 38.60 |  |
|  |  | 200 lx, 61 μW cm^-2^,  3,000 K  (WLED) |  | 0.98 | 78.75 | 32.68 | 19.73 |  |
| CsPbI_3_ | 1.69 | 522 lx, 168 μW cm^-2^,  2,956 K | 0.09 | 0.91 | 78 | 32.54 | 54.34 | [S4] |
|  |  | 106 lx, 33.84 μW cm^-2^,  2,956 K  (N/A) |  | 0.83 | 74 | 29.45 | 9.82 |  |
| FA_0.85_MA_0.15_PbI_3_ | 1.54 | 500 lx, 138 μW cm^-2^,  3,000 K | 0.09 | 0.93 | 80.92 | 39.31 | N/A | [S5] |
|  |  | 200 lx, 56 μW cm^-2^,  3,000 K |  | 0.90 | 74.59 | 38.31 |  |  |
|  |  | 500 lx, 138 μW cm^-2^,  3,000 K | 1 | 0.94 | 80.16 | 39.13 |  |  |
|  |  | 200 lx, 56 μW cm^-2^,  3,000 K |  | 0.90 | 79.86 | 36.84 |  |  |
|  |  | 500 lx, 138 μW cm^-2^,  3,000 K | 2.56 | 0.94 | 79.32 | 39.44 |  |  |
|  |  | 200 lx, 56 μW cm^-2^,  3,000 K  (N/A) |  | 0.90 | 74.59 | 36.84 |  |  |
| Cs_0.05_FA_0.70_MA_0.25_PbI_2.25_Br_0.75_ | 1.71 | 600 lx, 181 μW cm^-2^,  3,000 K | 0.09 | 1.03 | 80 | 41.09 | 74.43 | [S6] |
|  |  | 400 lx, 121 μW cm^-2^,  3,000 K |  | 1.02 | 79.62 | 40.31 | 48.69 |  |
|  |  | 200 lx, 60 μW cm^-2^,  3,000 K  (WLED) |  | 0.98 | 79.37 | 37.48 | 22.64 |  |
| N/A | 1.52 | 500 lx, 138 μW cm^-2^,  3,000 K | 0.07 | 0.94 | 80.91 | 39.03 | N/A | [S7] |
|  |  | 200 lx, 56 μW cm^-2^,  3,000 K  (DYSON, CD05) |  | 0.89 | 75.84 | 35.54 | N/A |  |
| Cs_0.05_(FA_0.75_MA_0.25_)_0.95_Pb(I_0.75_Br_0.25_)_3_ | 1.67 | 500 lx, 147 μW cm^-2^,  3,000 K | 0.07 | 1.05 | 82.10 | 39.53 | 56 | [S8] |
|  |  | 200 lx, 55 μW cm^-2^,  3,000 K  (N/A) |  | 1.01 | 81.57 | 38.55 | 21 |  |
| Cs_0.05_(FA_0.95_MA_0.05_)_0.95_Pb(I_0.95_Br_0.05_)_3_ | 1.55 | 500 lx, 157 μW cm^-2^,  3,000 K | N/A | 0.99 | 83.17 | 41.16 | N/A | [S9] |
|  |  | 200 lx, 65 μW cm^-2^,  3,000 K |  | 0.95 | 82.94 | 38.43 | N/A |  |
| Cs_0.05_FA_0.70_MA_0.25_PbI_2.25_Br_0.75_ | 1.71 | 600 lx, 172.7 μW cm^-2^, 2,950 K | 0.09 | 1.06 | 84.52 | 43.08 | 74.41 | This work |
|  |  | 400 lx, 115.3 μW cm^-2^, 2,950 K |  | 1.05 | 82.97 | 40.24 | 46.40 |  |
|  |  | 200 lx, 57.77 μW cm^-2^, 2,950 K  (WLED) |  | 1.02 | 82.83 | 40.94 | 23.65 |  |

**
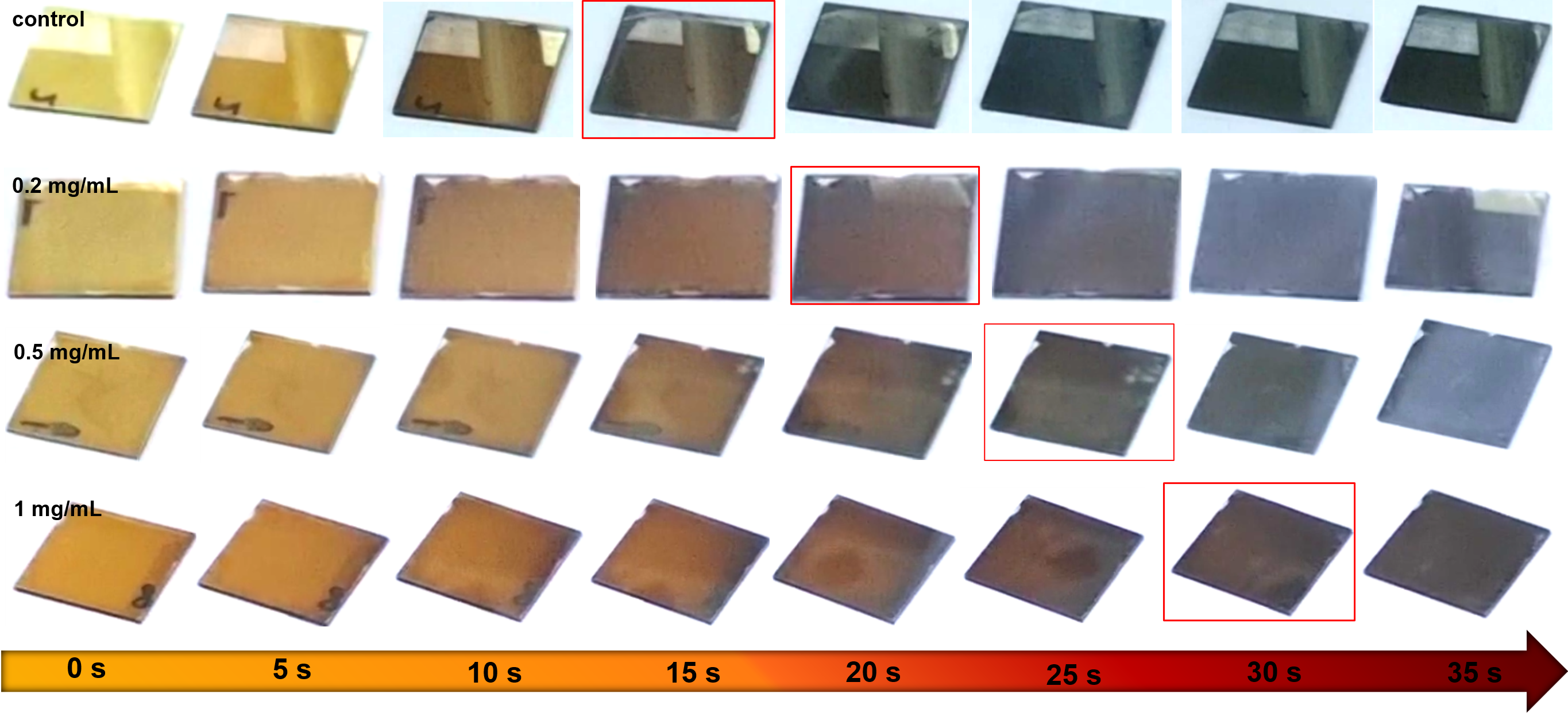
**

**Fig. S12** Time-dependent photographs of WBG-perovskite crystallization for different contents of PMMA in EA antisolvent by recording videos

**
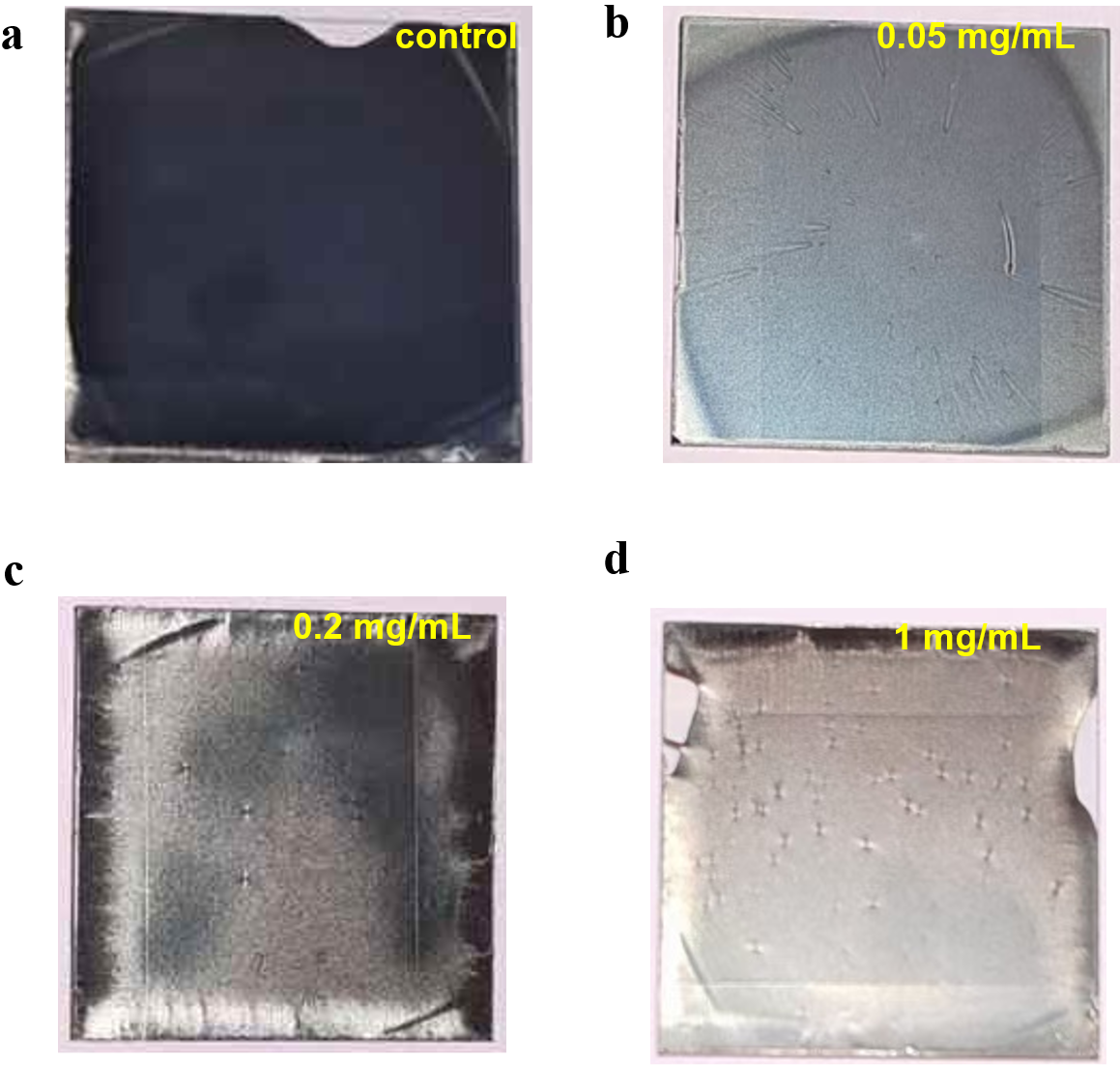
**

**Fig. S13** Photographs of surface structure of WBG-perovskite films incorporated PMMA. **a** Without PMMA, **b** 0.05 mg/mL, **c** 0.2 mg/mL and d 1 mg/mL

**
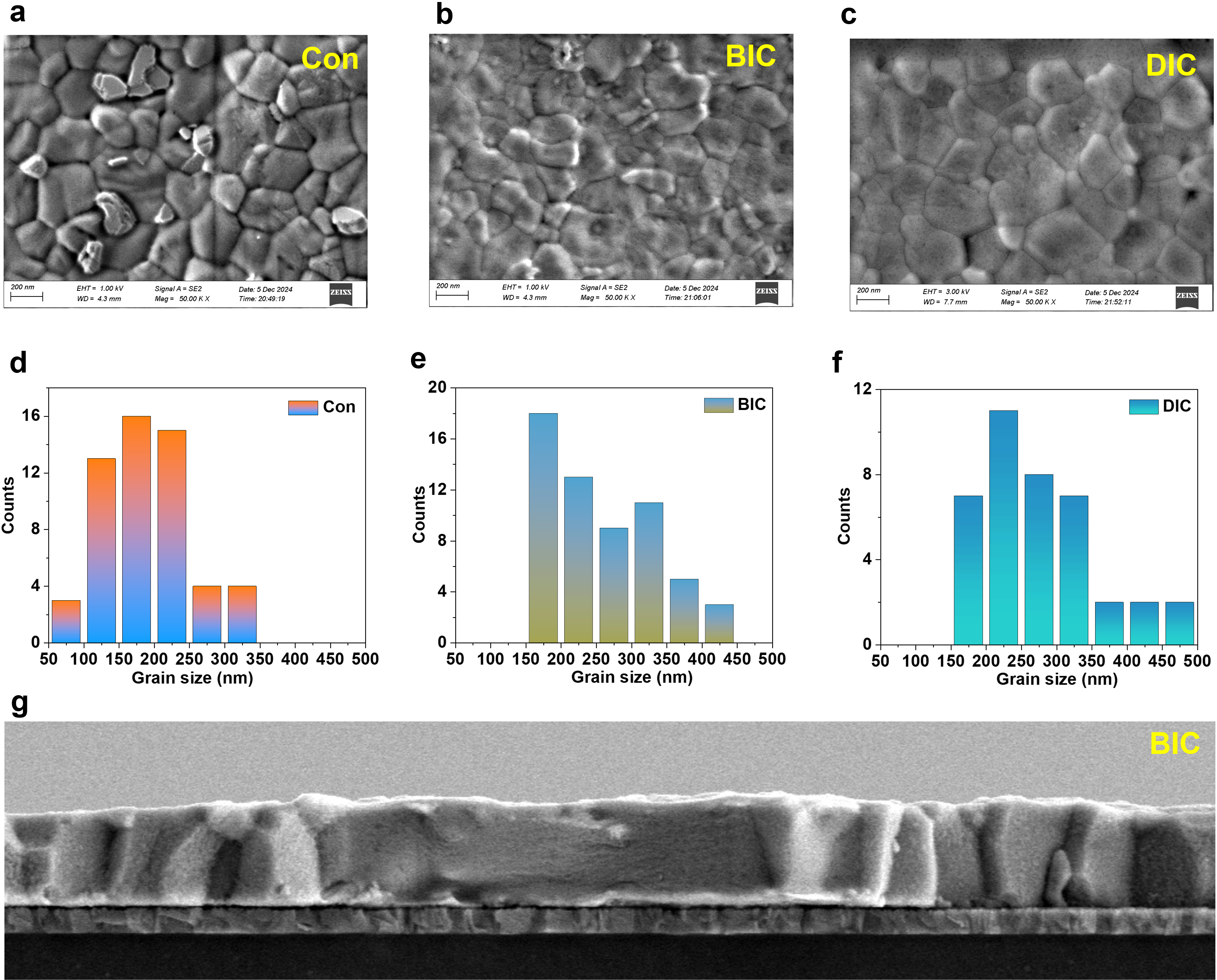
**

**Fig. S14 (a-c)** SEM images of surface morphologies and **(d-f)** corresponding crystalline grain size distribution histograms of the control, BIC- and DIC-WBG-perovskite films, and **(g)** cross-sectional SEM image of BIC-WBG-perovskite film

**
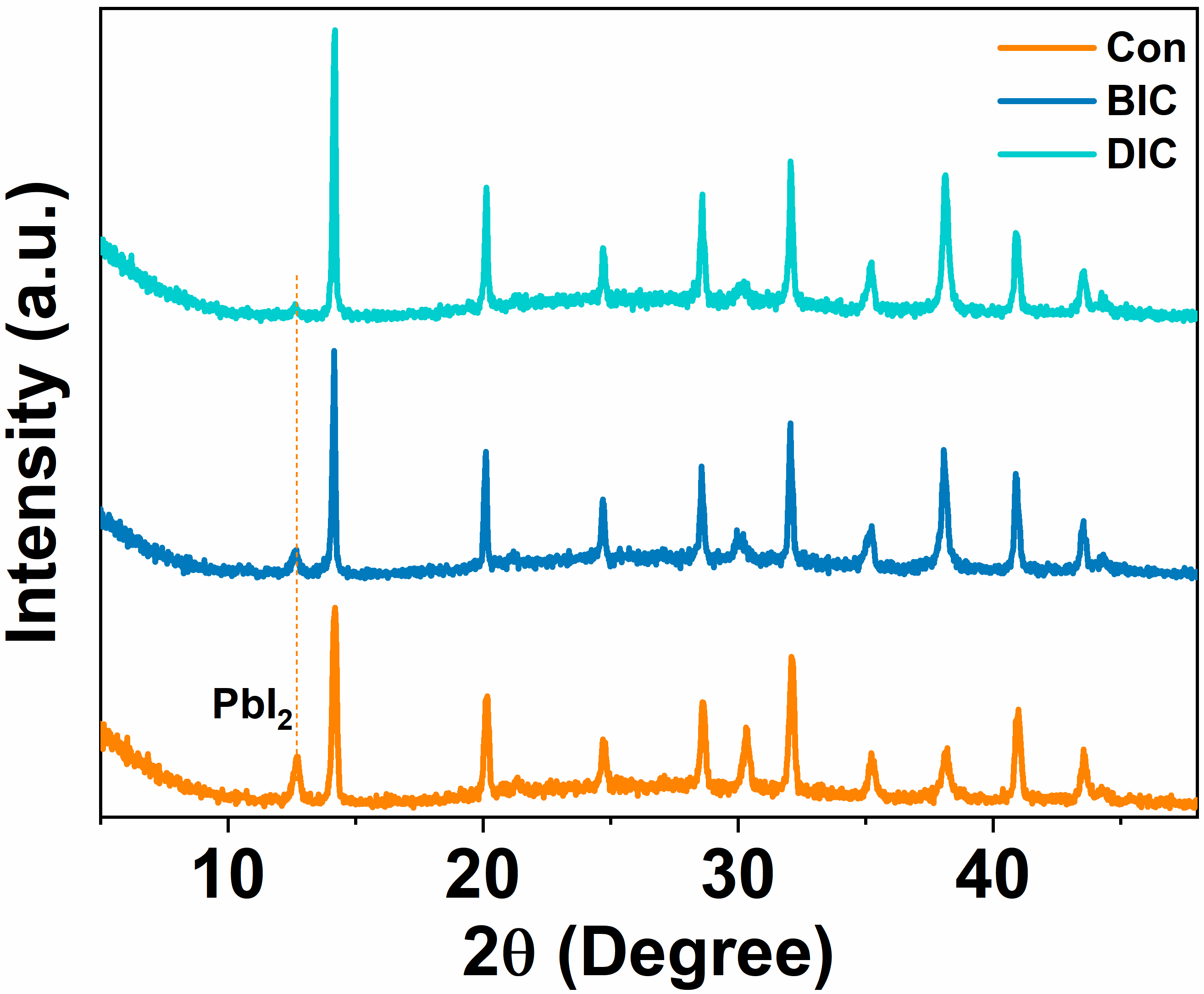
**

**Fig. S15** XRD patterns of control- (ITO/NiO*_x_*/PTAA/mp-Al_2_O_3_/WBG-perovskite), BIC- (ITO/NiO*_x_*/PTAA/mp-Al_2_O_3_-PMMA/WBG-perovskite) and DIC-perovskite films (ITO/NiO*_x_*/PTAA/mp-Al_2_O_3_-PMMA/WBG-perovskite-PMMA)

**
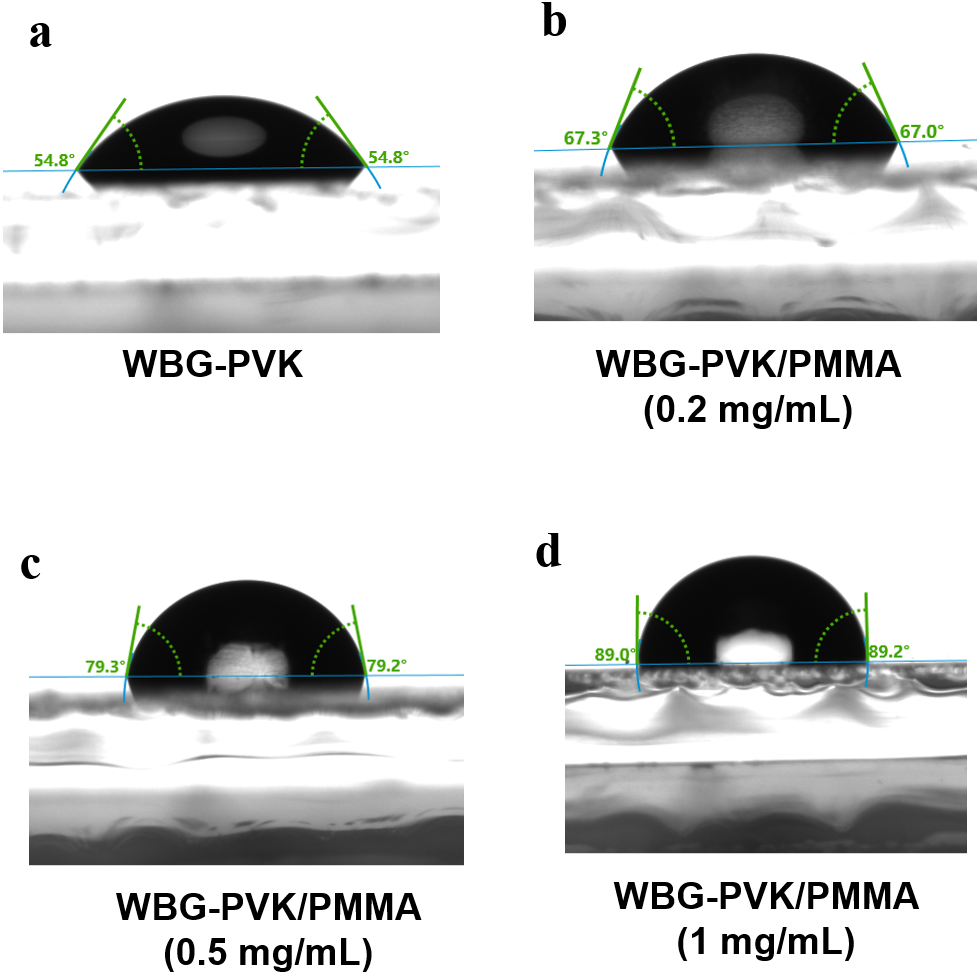
**

**Fig. S16** Water contact angles of the WBG-perovskite films incorporated different contents of PMMA: **a** without PMMA, **b** 0.2 mg/mL, **c** 0.5 mg/mL and **d** 1 mg/mL.


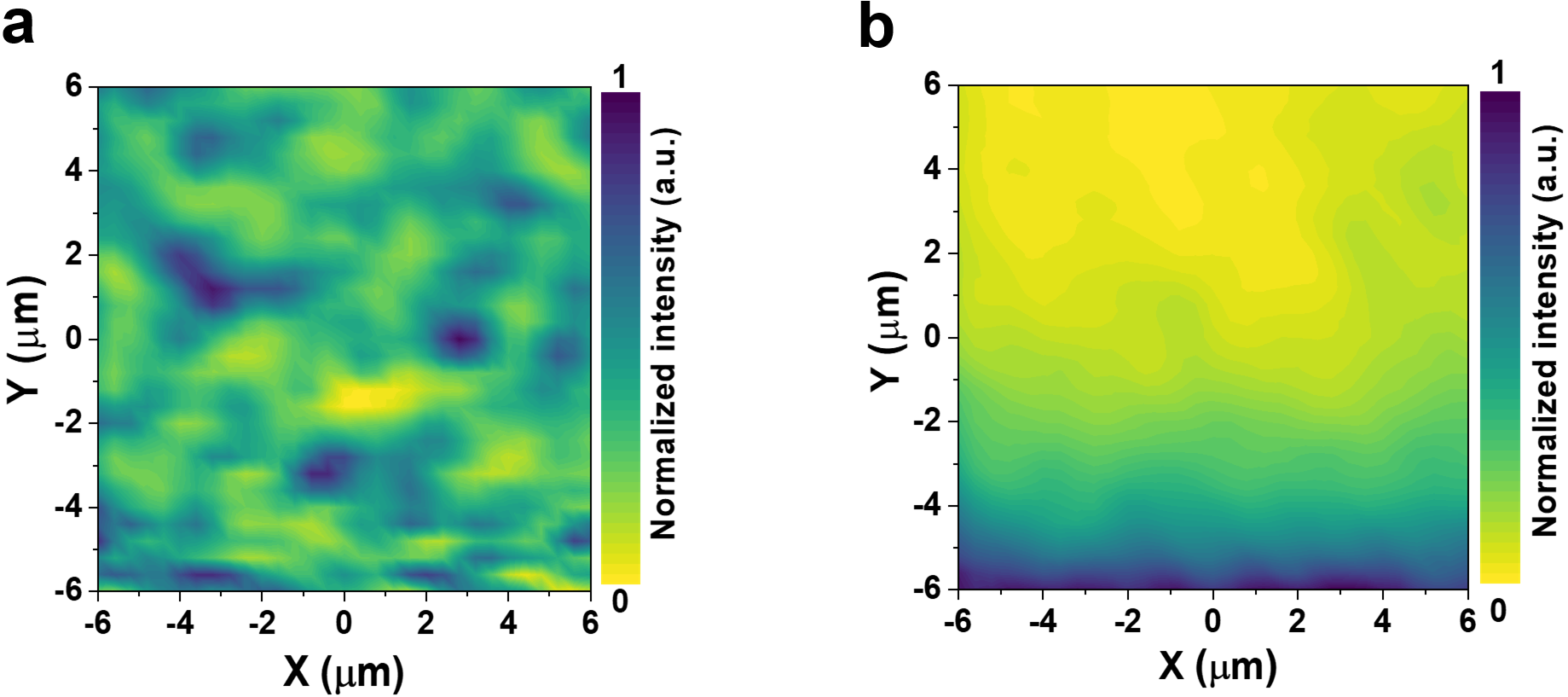


**Fig. S17** PL mapping images for control (ITO/NiO*_x_*/PTAA/Al_2_O_3_/WBG-perovskite) and DIC-based WBG-perovskite films (ITO/NiO*_x_*/PTAA/Al_2_O_3_-PMMA/WBG-perovskite-PMMA)


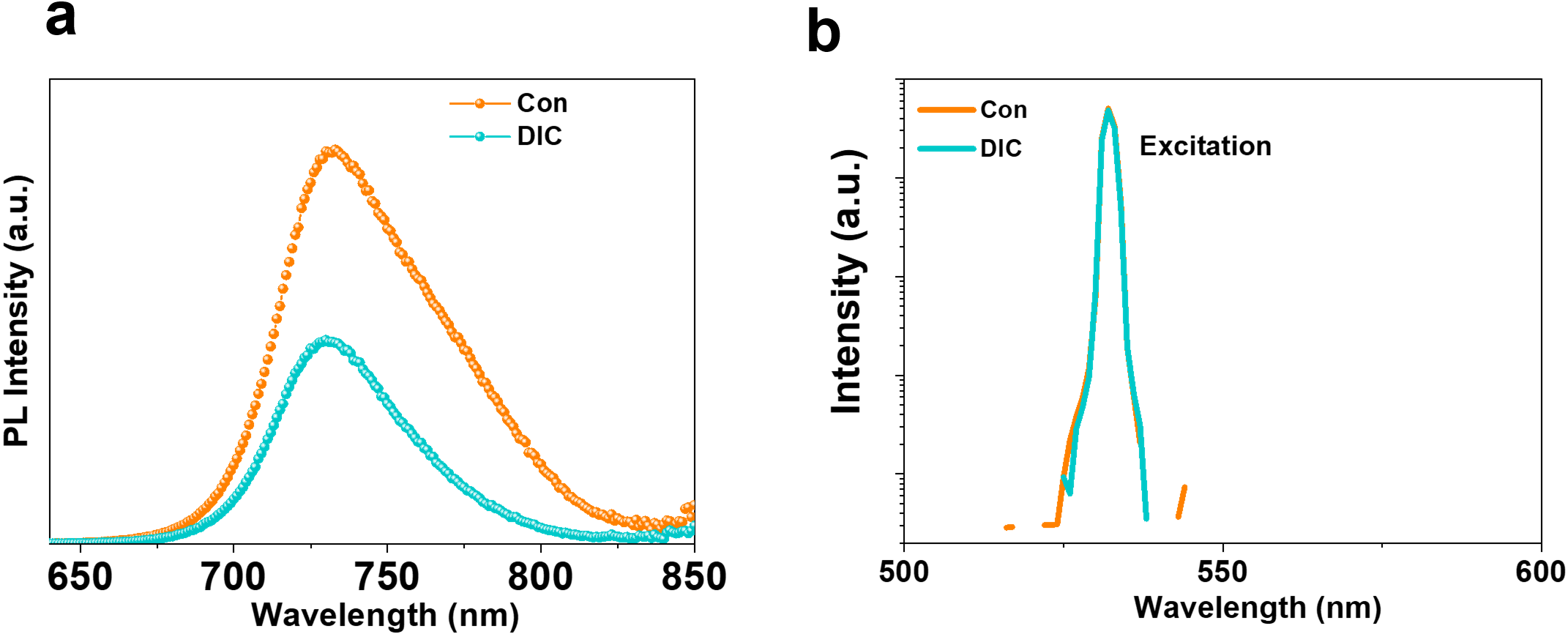


**Fig. S18** Steady-state PL and PLQY with an excitation of 532 nm for control (ITO/NiO*_x_*/PTAA/Al_2_O_3_/WBG-perovskite) and DIC-based WBG-perovskite films (ITO/NiO*_x_*/PTAA/Al_2_O_3_-PMMA/WBG-perovskite-PMMA)

**
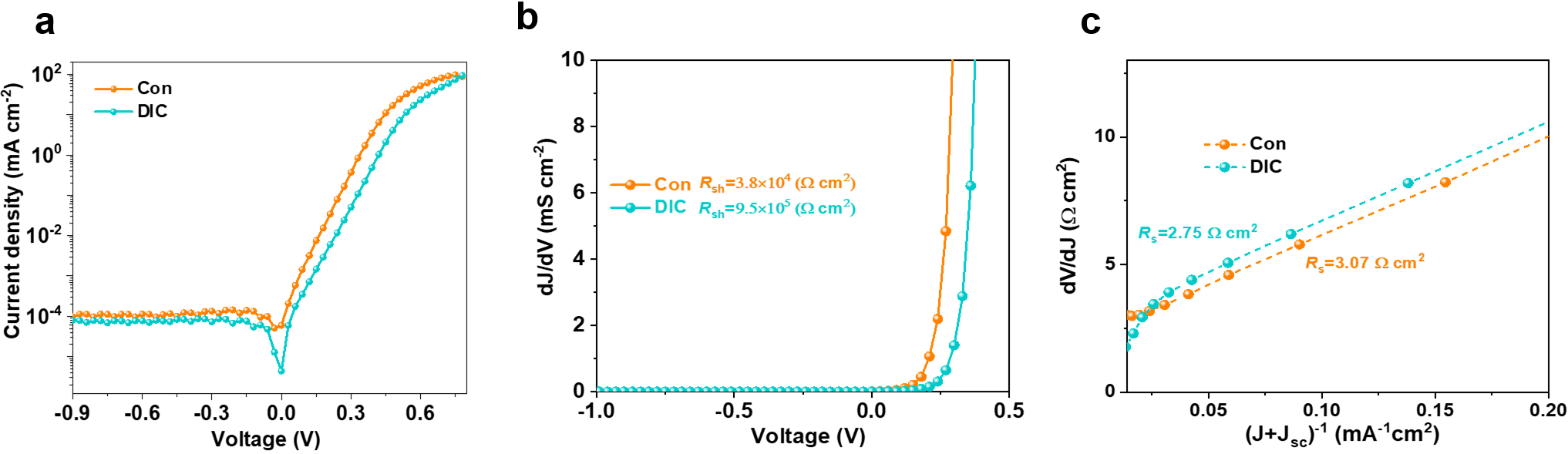
**

**Fig. S19** The electrical conductivity characteristics. **a** Dark *J-V* curves, **b** the *dJ/dV* with the fit used to determine *R*_sh_, **c** the *dV/dJ* with the fit used to determine *R*_s_ for control and DIC-based devices


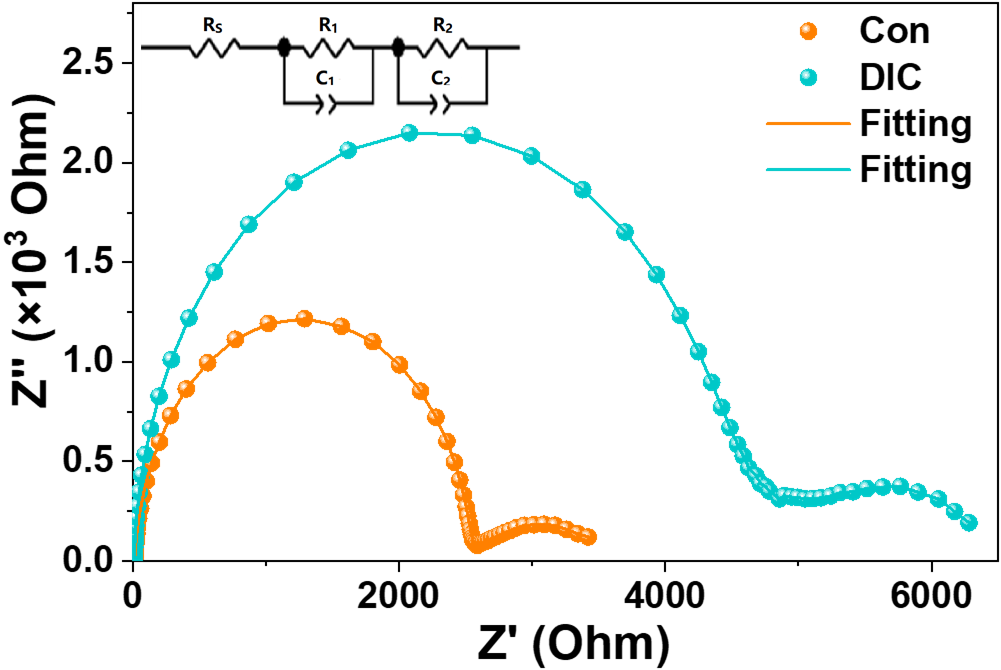


**Fig. S20** EIS Nyquist plots measured by the electrochemical impedance spectra (EIS) of the control- and DIC-devices under dark conditions with an bias voltage of 1.1 V

**Table S10** Impedance parameters of control and DIC-devices obtained from fitting EIS data

| **Device** | ***R*_s_ (Ω)** | ***R*_1_ (Ω)** | ***C*_1_ (×10^-8^ F cm^-2^ sn^-1^)** | ***R*_2_ (Ω)** | ***C*_2_ (×10^-5^ F cm^-2^ sn^-1^)** |
| --- | --- | --- | --- | --- | --- |
| Con | 15.08 | 2496 | 1.286 | 148.91 | 23.768 |
| DIC | 11.50 | 4413 | 1.087 | 1424 | 3.116 |

**
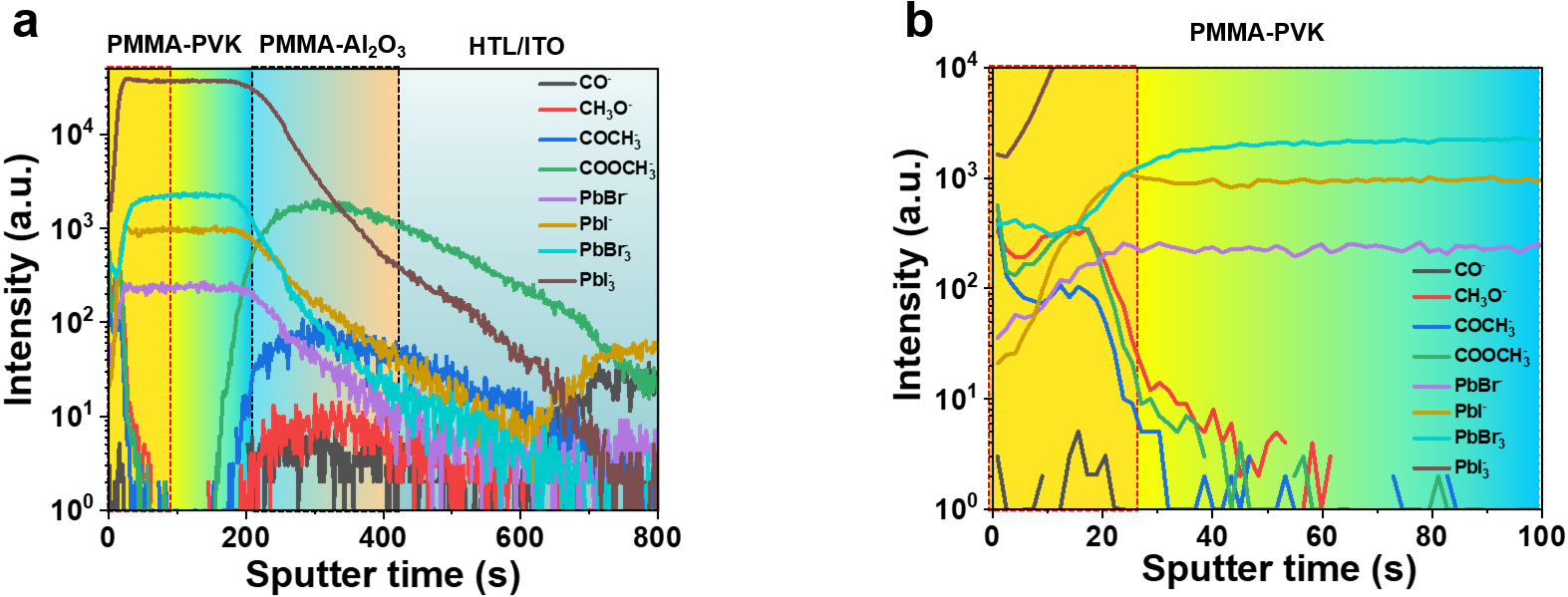
**

**Fig. S21** ToF-SIMS depth profile for ITO/HTL/PMMA-Al_2_O_3_/PMMA-PVK at 0-800 s

**Supplementary References**

1. X. He, J. Chen, X. Ren, L. Zhang, Y. Liu et al., 40.1% record low-light solar-cell efficiency by holistic trap-passivation using micrometer-thick perovskite film. Adv. Mater. **33**(27), 2100770 (2021). <https://doi.org/10.1002/adma.202100770>
2. C.-H. Chen, Z.-H. Su, Y.-H. Lou, Y.-J. Yu, K.-L. Wang et al., Full-dimensional grain boundary stress release for flexible perovskite indoor photovoltaics. Adv. Mater. **34**(16), e2200320 (2022). <https://doi.org/10.1002/adma.202200320>
3. C. Zhang, M. He, S. Wu, Y. Gao, M. Ma et al., Occlusal architecture of the buried interface enables record-efficiency flexible perovskite photovoltaic modules with enhanced in-plane bending mechanical endurance. Adv. Funct. Mater. **34**(19), 2313910 (2024). <https://doi.org/10.1002/adfm.202313910>
4. K.-L. Wang, H. Lu, M. Li, C.-H. Chen, B.Z. Ding- et al., Ion-dipole interaction enabling highly efficient CsPbI(3) perovskite indoor photovoltaics. Adv. Mater. **35**(31), e2210106 (2023). <https://doi.org/10.1002/adma.202210106>
5. Y. Li, T. Nie, X. Ren, Y. Wu, J. Zhang et al., *In situ* formation of 2D perovskite seeding for record-efficiency indoor perovskite photovoltaic devices. Adv. Mater. **36**(1), e2306870 (2024). <https://doi.org/10.1002/adma.202306870>
6. Q. Ma, Y. Wang, L. Liu, P. Yang, W. He et al., One-step dual-additive passivated wide-bandgap perovskites to realize 44.72%-efficient indoor photovoltaics. Energy Environ. Sci. **17**(5), 1637–1644 (2024). <https://doi.org/10.1039/d3ee04022d>
7. C. Liu, T. Yang, W. Cai, Y. Wang, X. Chen et al., Flexible indoor perovskite solar cells by *in situ* bottom-up crystallization modulation and interfacial passivation. Adv. Mater. **36**(24), e2311562 (2024). <https://doi.org/10.1002/adma.202311562>
8. C.-H. Chen, X.-Y. He, R.-H. Qin, K.-L. Wang, L. Huang et al., Reliable perovskite indoor photovoltaics for self-powered devices. Natl. Sci. Rev. **12**(8), nwaf242 (2025). <https://doi.org/10.1093/nsr/nwaf242>
9. J. Jin, Z. Zhu, Y. Ming, Y. Zhou, J. Shang et al., Spontaneous bifacial capping of perovskite film for efficient and mechanically stable flexible solar cell. Nat. Commun. **16**(1), 90 (2025). <https://doi.org/10.1038/s41467-024-55652-6>
